# Dynamics of an RNase H‑Responsive Tetrahedral DNA Nanostructure for Efficient Intracellular microRNA Inhibition

**DOI:** 10.1021/acs.bioconjchem.5c00563

**Published:** 2026-04-22

**Authors:** Ana S. G. Martins, Sara D. Reis, Ruxandra Baboi, Francisco Furlan, João Cortinhas, Filomena A. Carvalho, Nuno C. Santos, Jonathan Bath, Erik Benson, Ana P. Pêgo, Pedro M. D. Moreno

**Affiliations:** † i3S–Instituto de Investigação e Inovação em Saúde and INEB–Instituto de Engenharia Biomédica, Universidade do Porto, Rua Alfredo Allen 208, 4200-135 Porto, Portugal; ‡ FEUP–Faculdade de Engenharia da Universidade do Porto, 4200-465 Porto, Portugal; § GIMM–Gulbenkian Institute for Molecular Medicine, 1649-028 Lisbon, Portugal; ∥ Faculdade de Medicina, Universidade de Lisboa, 1649-028 Lisbon, Portugal; ⊥ Kavli Institute for Nanoscience Discovery, 6396 University of Oxford, Dorothy Crowfoot Hodgkin Building, Oxford OX1 3QU, United Kingdom; # Science for Life Laboratory (SciLifeLab), Department of Microbiology, Tumor and Cell Biology, Karolinska Institutet, 171 65 Solna, Sweden; ∇ ICBAS–Instituto de Ciências Biomédicas Abel Salazar, Universidade do Porto, 4050-313 Porto, Portugal

## Abstract

Tetrahedral DNA nanostructures (TDNs) are emerging as next-generation platforms for delivering therapeutic oligonucleotides. This study introduces a novel strategy embedding antisense oligonucleotide (ASO) sequences directly within the structural framework of TDNs, not requiring external extensions as hybridization handles and preserving flexibility for functionalization. The integration of a gapmer-based design enables structural reconfiguration upon cellular delivery, promoting ASO accessibility and efficient target engagement. To validate this approach, we engineered a TDN-gapmer targeting microRNA-21 (miR-21), a dysregulated biomarker linked to glioblastoma. Without requiring transfection agents, TDN-gapmer demonstrated autonomous delivery capacity into glioblastoma cells, leading to robust miR-21 inhibition. Mechanistic studies revealed that the integrated gapmer can potentially recruit ribonuclease (RNase) H, facilitating RNA cleavage and enhancing target suppression. Coarse-grained modeling provided a detailed view of the predicted structural transitions and thermodynamic parameters that demonstrate that cleavage initiates toehold formation, enabling strand displacement and potential catalytic reconfiguration of the nanostructures. The TDN-gapmer demonstrated stability in serum, withstanding degradation while maintaining its therapeutic potential. The ability to integrate active sequences into the structural framework increases availability for further potential multifunctionalization. This innovative TDN design establishes a versatile and transformative platform with promising implications for precision RNA-targeting therapeutics.

## Graphical abstract

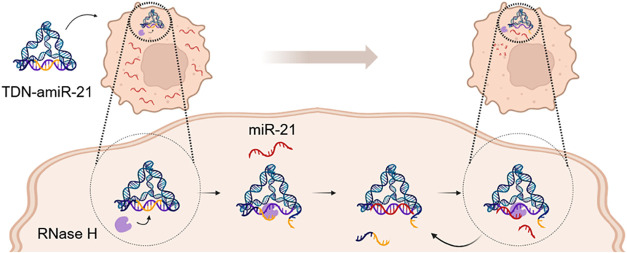

## 1 Introduction

Structural DNA and RNA nanotechnology provides an interesting exploratory avenue for the design of nucleic acid nanostructures with different biological activities. Being intrinsically composed of nucleic acids, these nanostructures enable a seamless integration of oligonucleotide therapeutics (OTs), functioning as delivery vehicles of these bioactive drugs.^1^

A particularly interesting DNA nanostructure is represented by the tetrahedral DNA nanostructure (TDN), first reported by Turberfield et al.^2^ This is one of the simplest and smallest self-assembled three-dimensional (3D) DNA nanostructure, being formed by a minimum of 4 single-stranded oligonucleotides, produced in high yields and with significant structural stability in physiological conditions.^3,4^ In addition, this structural geometry provides increased cell membrane interactions and cell uptake when compared to unstructured double-stranded DNA (dsDNA).^5^

Generally, loading functional oligonucleotides, OTs, to these nanostructures has been mainly accomplished through extending the oligonucleotide sequences that compose the TDN framework, resulting in dangling single-strand extensions at 3′ or 5′ with the OT^6–8^ or complementary sequences to the OT,^1,9–11^ or looped single-strand sequences protruding from the sides of the nanostructure.^12,13^

An alternative approach would involve integrating the functional OTs within the framework of TDN. This would allow potentially decreasing the number and length of structural oligonucleotide sequences used per nanostructure. More importantly, this would leave the structure with more functionalization sites available for further ligand multifunctionalization, for instance, by hybridization to TDN extensions, a method applied in our previous work in the engineering of a multifunctional TDN.^10^ A challenge from an integrated design configuration is that the OT sequence is not readily accessible to bind the target RNA. Thus, strategies to promote the intracellular release or exposure of the OTs need to be developed.

Here, we present a novel design of TDN with an antisense oligonucleotide (ASO) sequence included in one of the TDN framework strands, at a structural edge, thus being fully integrated in the core framework sequence. A gapmer design is used both as the functional antisense sequence (therapeutic effect) and as the mechanism to trigger the intracellular exposure of the active antisense region, finally leading to target RNA inhibition. As a proof of concept, we tested an anti-microRNA-21 (anti-miR-21) gapmer sequence to inhibit microRNA-21 (miR-21) reported to be upregulated in several cancers,^14^ namely glioblastoma and bone osteosarcoma.^15,16^ With this new TDN-gapmer, we were able to demonstrate, without the assistance of transfection agents, engagement of our functional nanostructure with a target microRNA (miR-21), leading to its cleavage *in vitro* and inhibition in a glioblastoma cell line model system.

## 2 Results

### 2.1 Design of Tetrahedral DNA Nanostructure Integrating Anti-MicroRNA-21 (TDN-amiR-21)

For the development of a novel design of a TDN with an integrated gapmer anti-miRNA sequence, a gapmer anti-miR-21 sequence was encoded within one of the TDN strands, forming the core framework structure of a TDN. In the complementary region to the gapmer sequence, a short unmodified RNA sequence was used in order to form a DNA:RNA heteroduplex. This is expected to trigger the recruitment of the RNase H enzyme to cleave the RNA sequence, promoting the exposure of the gapmer and increasing its accessibility to bind to its target RNA (miR-21) (Figure 1A). The target RNA can then remain sterically blocked or be cleaved by RNase H, initiating a catalytic cleavage process (Figure 1B,C). In the process of the TDN sequence design, we envisioned a compromise between the stability of TDN-amiR-21 upon RNase H-mediated cleavage and accessibility of the gapmer to its target RNA.

**Figure 1:**
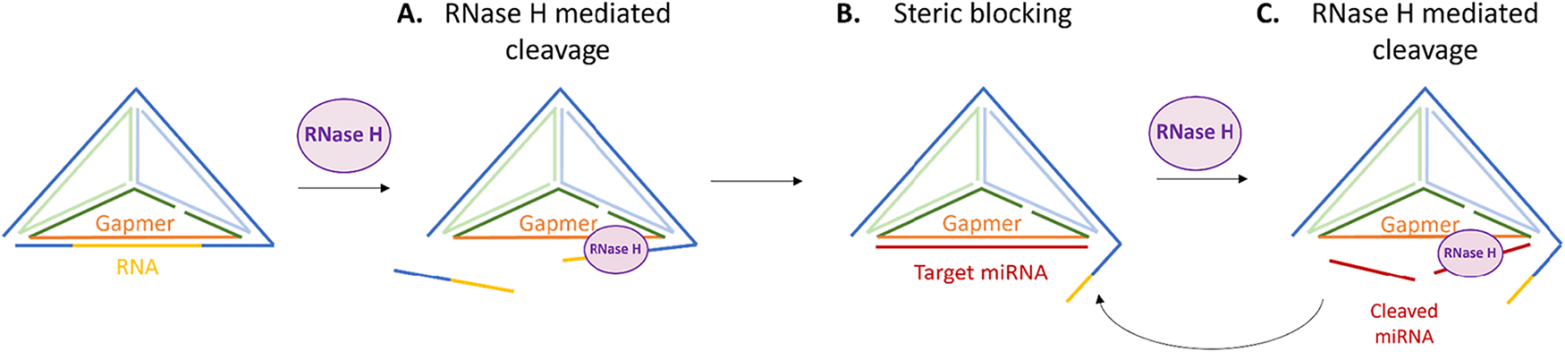
Proposed mechanism of action of antisense gapmer integrated in TDN. (A) The hybrid gapmer–RNA is expected to be recognized by RNase H, which will trigger RNA cleavage. (B) Target RNA with full complementarity to the gapmer sequence will hybridize with high affinity, possibly displacing fragments of the previous RNA sequence. This will sterically block the target RNA within TDN. (C) RNase H can be recruited, repeating the cleavage process from step A.

Prior to selecting the gapmer design to introduce in the TDN sequence, two patterns of nucleotide modifications of an anti-miR-21 gapmer sequence were explored in order to select a pattern that could elicit high *in vitro* inhibitory activity. The gapmer designs consisted of a DNA central block of 8 nucleotides, flanked by 2′-O-methyl (2′-O-Me) and locked nucleic acid (LNA) modified nucleotides, differing in the number and position of the LNA-modified nucleotides (Figure S1). Both 2′-O-Me and LNA nucleotides contribute to an increase in the binding affinity to the target RNA, which generally leads to an increase in oligonucleotides potency, with LNA presenting the highest affinity increase.^17,18^ The selection of the alternated pattern 2′-O-Me and LNA (gapmer A) was adapted from a study identifying this pattern as the most potent design tested when applied to a fully modified anti-miR-21, with steric blocker activity, avoiding RNase H degradation.^17^ The selection of the pattern with four consecutive LNAs in each end followed by 2′-O-Me (gapmer B), was based on a study exploring different chemical modifications of gapmer ASOs, identifying potent 14-nt gapmers with two consecutive LNAs at each end, followed by one 2′-O-Me, and including a DNA central block of 8 nucleotides.^19^ As control, a fully 2′O-Me modified sequence was used in this study, including three phosphorothioate (PS) linkages at each end, with reported activity as a steric blocker oligonucleotide.^17,20^ Three PS linkages were also included at each end of the gapmers tested in this study to confer resistance to nucleases. These gapmer designs were applied to a 20-nucleotide (nt) anti-miR-21 sequence, with full complementarity to the 22-nt miR-21.

The RNase H recruitment activity by anti-miR-21 gapmers was then verified by polyacrylamide gel electrophoresis (PAGE) (Figure S2). Gapmer B provided a faster recruitment, assessed by quantification of the dissociated gapmer as a function of incubation time, indicating higher efficiency. Subsequently, the anti-miR-21 gapmers inhibitory activity was evaluated *in vitro* using a luciferase reporter system in U2OS cell line, which endogenously expresses high levels of miR-21 (Figure S3). The efficiency of the inhibitory activity of anti-miR-21 sequences was measured through an increase in luciferase activity (luminescence release). Both gapmers showed microRNA inhibitory activity with a similar efficiency, which was higher than the inhibitory activity found for the control anti-miR-21 oligonucleotide sequence at the highest concentration tested. Given the fast RNase H recruitment in the RNase H assay by PAGE (Figure S2B), the anti-miR-21 gapmer B was the selected sequence to be incorporated in TDN-amiR-21.

The TDN sequences were then designed, encoding the selected anti-miR-21 gapmer sequence within one of the TDN strands (S1-amiR-21 strand; Table S2). For the design of the overall TDN component strands, we used NUPACK software, predefining the gapmer and its complementary sequence, following the steps in Figure S4. For the design of a TDN with a scramble sequence (TDN-scr), only the gapmer and its complementary sequence were changed in relation to TDN-amiR-21 sequences.

### 2.2 Characterization of TDN-amiR-21 Assembly

TDN-amiR-21 was characterized by different techniques, as shown in Figure 2. The assembly of TDN-amiR-21 was analyzed by PAGE, comparing the migration of individual TDN-amiR-21 composing strands and assembled TDN-amiR-21 (Figure 2A). Each individual nanostructure strand migrated as the 25–50 base pairs (bp) dsDNA marker, whereas the self-assembled monomeric TDN-amiR-21 migrated slower, close to the 150–200 bp dsDNA marker. In addition, some higher-molecular-weight (*M*_W_) bands were observed with lower intensity, representing concatemers, mis-assembled DNA sequences that are inevitably formed during the DNA self-assembly of structures with nick defects.^21^ The monomeric TDN-amiR-21 was obtained with an assembly yield of 88.2% ± 2.6% (quantification by gel densitometry from 4 independent gels), which is in line with TDN assembly yields verified in our own previous work.^10^

**Figure 2:**
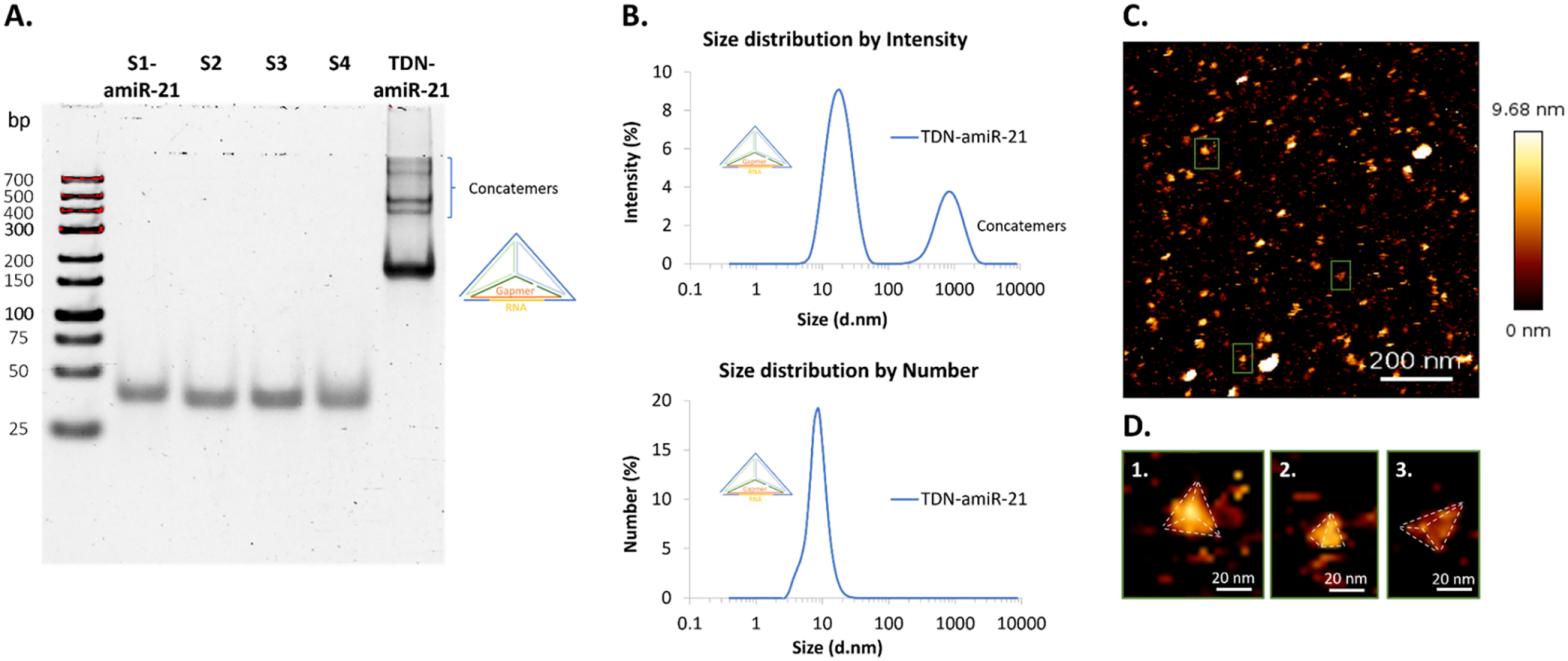
Characterization of TDN-amiR-21. (A) PAGE analysis of TDN-amiR-21 and individual TDN-amiR-21 strands (S1-amiR-21, S2, S3, S4). 6% (w/v) resolving native gel, supplemented with 5 mM MgCl_2_. (B) DLS analysis of TDN-amiR-21. Top plot represents the intensity-weighted distribution (Z-average hydrodynamic diameter = 23.9 nm ± 5.9 nm and polydispersity index (PdI) = 0.56 ± 0.18), and the bottom plot represents the number-weighted distribution (number-averaged hydrodynamic diameter = 8.8 nm ± 1.0 nm). The plots are from one representative experiment, with 3 technical replicates. The average values correspond to 3 independent experiments, 3 technical replicates each. (C) Illustrative AFM image, color-coded according to the height of the detected nanostructures (height scale bar in the right). TDN-amiR-21 sample analyzed by AFM after self-assembly, with no further purification. (D) Selected regions from (C) zoomed 10×, with dashed lines representing possible tetrahedral outlines.

Dynamic light scattering (DLS) measurements confirmed the self-assembly of TDN-amiR-21 mainly into monomeric structures, with concatemers present in a lower percentage (intensity-weighted distribution). Since nanoparticles in solution scatter the incident light with an intensity proportional to the sixth power of their radii, the larger size nanostructures (concatemers) are detected with much higher intensity than smaller structures.^22^ Thus, the conversion from intensity- to number-weighted distribution is considered to compensate for the differences in light scattering intensity detection in bimodal distributions.^23^ A mean hydrodynamic diameter of approximately 9 nm was obtained from the number-weighted distribution, in accordance with previously reported measurements^10,24–26^ (Figure 2B).

The topology of TDN-amiR-21 was examined by atomic force microscopy (AFM). Most nanostructures consistent with monomeric TDN show triangular morphology with apparent heights within the expected theoretical range (5 – 6 nm) and consistent with previously reported dimensions (5 – 9 nm). (Figures 2C,D and S5).^10,27–29^ The brighter objects with the largest dimensions likely represent the concatemers resultant from self-assembly, in agreement with PAGE analysis.

Next, the stability of the TDN-amiR-21 and TDN-amiR-21 with fully PS-modified gapmer sequence (TDN-amiR-21_PS) was evaluated in 10% (v/v) fetal bovine serum (FBS), by examining their degradation profile up to 48 h (Figures 3 and S6). Few differences were observed from 0 to 48 h incubation in FBS, indicating low degradation up to 48 h, in accordance with results from our previous TDN study.^10^ When TDN-amiR-21 is compared in the presence *versus* absence of serum, some degradation is detected immediately after FBS addition, which then stabilizes with continued incubation time. Analyzing the free single strands, we verified a partial degradation of the S4 strand into two lower-*M*_W_ bands upon adding FBS, indicating possible RNA sequence degradation, more susceptible to nucleases degradation than DNA. After 24 h serum incubation, free single strands were rapidly degraded in opposition to the strands self-assembled in TDN-amiR. In free S1-amiR-21_PS, a well-defined lower-*M*_W_ band appears after 24 h serum incubation, with the size corresponding to the free gapmer anti-miR-21, indicating degradation of the regions flanking the gapmer, composed of natural DNA bases. In S1-amiR-21 without PS, the lower-*M*_W_ band is barely visible, indicating that PS modifications are those most contributing to the increase in nuclease resistance in that region. The free gapmer anti-miR-21, which includes PS modifications in each flank, showed a high resistance to nuclease degradation up to 24 h in serum.

**Figure 3:**
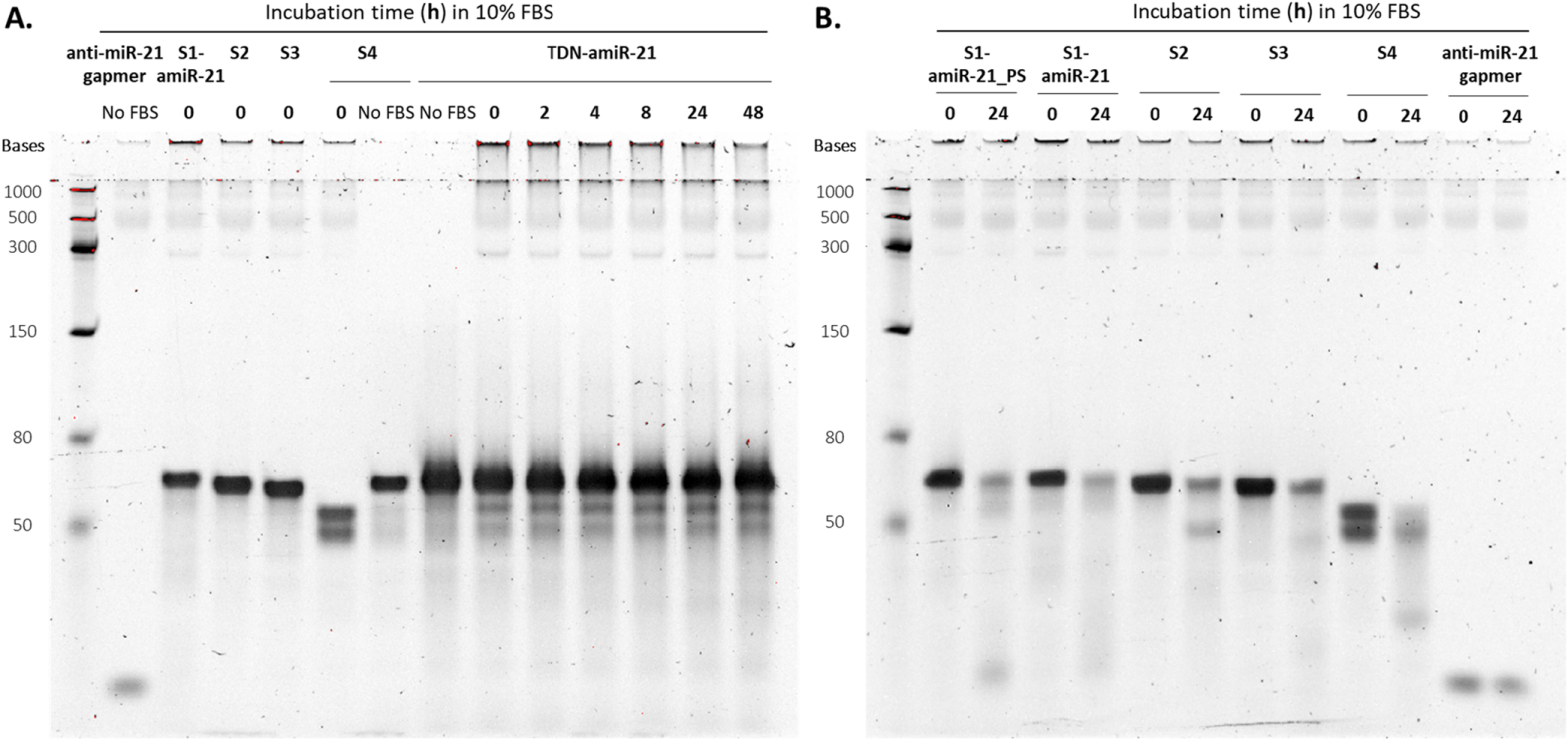
Denaturing PAGE analysis of TDN-amiR-21 incubated up to 48 h in DMEM with 10% FBS (noninactivated) (A). Single strands that compose TDN-amiR-21 and anti-miR-21 gapmer were analyzed as experimental controls to determine differences in stability that can contribute to the overall TDN-amiR-21 stability and incubated for 0 and 24 h (B). 8% (w/v) resolving denaturing gel, with 8 M urea.

### 2.3 RNase H Recruitment by TDN-amiR-21

RNase H recruitment efficiency of TDN-amiR-21 was assessed by PAGE (Figure 4). By native PAGE (Figure 4A), we detected differences in the migration of TDN-amiR-21 before and after incubation with RNase H, with a faster migration after RNase H. This suggests a reduction in the *M*_W_ of TDN-amiR, indicating cleavage of the RNA strand. Upon mixing TDN-amiR-21 with miR-21, we observed a similar change in the migration of TDN-amiR-21 after incubation of RNase H and, importantly, we additionally observed the disappearance of the miR-21 band, which could be detected as a lower-*M*_W_ band before addition of RNase H. Overall, this result demonstrates that TDN successfully enabled the recruitment of RNase H and miR-21 cleavage.

**Figure 4:**
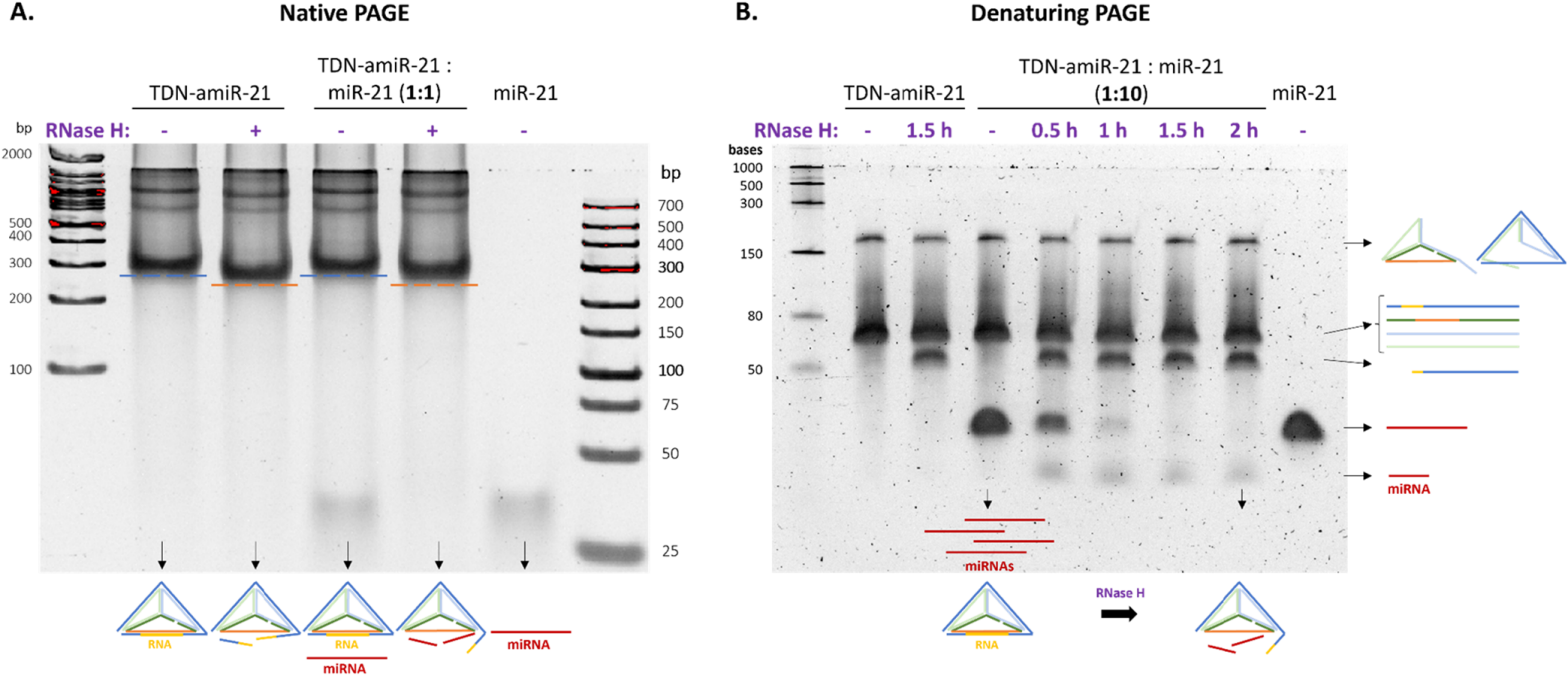
RNase H assay by PAGE analysis. (A) Native PAGE analysis of TDN-amiR-21 and a mix of TDN-amiR-21: miR-21 at a ratio of 1:1, incubated for 1.5 h with or without RNase H. 8% (w/v) resolving native gel, supplemented with 3 mM MgCl_2_. (B) Denaturing PAGE analysis of TDN-amiR-21 and a mix of TDN-amiR-21:miR-21 at a ratio of 1:10, incubated in the absence or presence of RNase H up to 2 h. The controls TDN-amiR-21 and a TDN-amiR-21:miR-21 (1:10) without RNase H were incubated for 1.5 and 2 h, respectively, in parallel with the samples with RNase H. 8% (w/v) resolving denaturing gel, with 8 M urea.

To test the TDN-amiR-21 catalytic activity, an excess of miR-21 (10×) was added to TDN-amiR-21, incubated with RNase H at different time points, and analyzed by denaturing PAGE (Figure 4B). Each fully denatured strand that composes the TDN (64 nt or 66 nt long) is observed as a single size band migrating at an intermediate position between the 50 nt and 80 nt bands from the ladder. A less intense band, with a higher *M*_W_, is also visible due to an incomplete denaturation of the strands. After incubation with RNase H, a band is formed with a lower *M*_W_ than single TDN strands, but higher than 50 nt. This is in accordance with the size of the S4 strand after RNA cleavage, which should be around 51 to 57 nt, since the 10-nt-long RNA strand is placed starting at the seventh nucleotide of S4 and is hybridized with nonmodified DNA nucleotides for 6 nt further. Thus, a main cut with a maximum size of 7–13 nt should be expected. After mixing TDN-amiR-21 with 10× miR-21, without RNase H, we observe a lower-*M*_W_ band due to the excess of miR-21. After 2 h incubation with RNase H, the miR-21 band completely disappears, with a lower-*M*_W_ band being formed, which indicates cleaved miR-21. Thus, TDN-amiR-21 can support the potential catalytic cleavage of RNA.

Additionally, the cleaved miRNA was observed to be in the range of 13 nucleotides by PAGE analysis, using specific ssDNA size markers for comparison of gel migration (Figure S7). This shows that the main RNase H-mediated cleavage site occurs at the 3′-end of the RNA sequence within the nonmodified DNA-RNA duplex region, just before the first base pair containing a 2′-O-Me modified nucleotide. Thus, the gapmer anti-miR-21 integrated in the tetrahedron structure induces a similar cleavage pattern to that previously described by Lima and Crooke, toward the 3′ end of the RNA at RNA-DNA gapmer substrates.^30^ In this case, this corresponds to the 13 nt from the 5′-end of the S4 strand, which includes the RNA strand in the DNA-RNA hybrid duplex.

### 2.4 Modeling

TDN was designed to induce multiple cleavages of its cognate miRNA. The gapmer was shown above to direct an asymmetric cleavage of the miRNA-mimic strand, generating a 13 nt fragment (from the 5′end of S4 before cleavage) and a 7 nt fragment (still integrated in the TDN; 5′-end of the S4 strand after cleavage). Spontaneous dissociation of the 7 nt strand is expected to reveal a single-stranded toehold, which allows the target miRNA to bind and displace the longer 13 nt fragment, completing the potential catalytic cycle (Figure 5).

**Figure 5:**
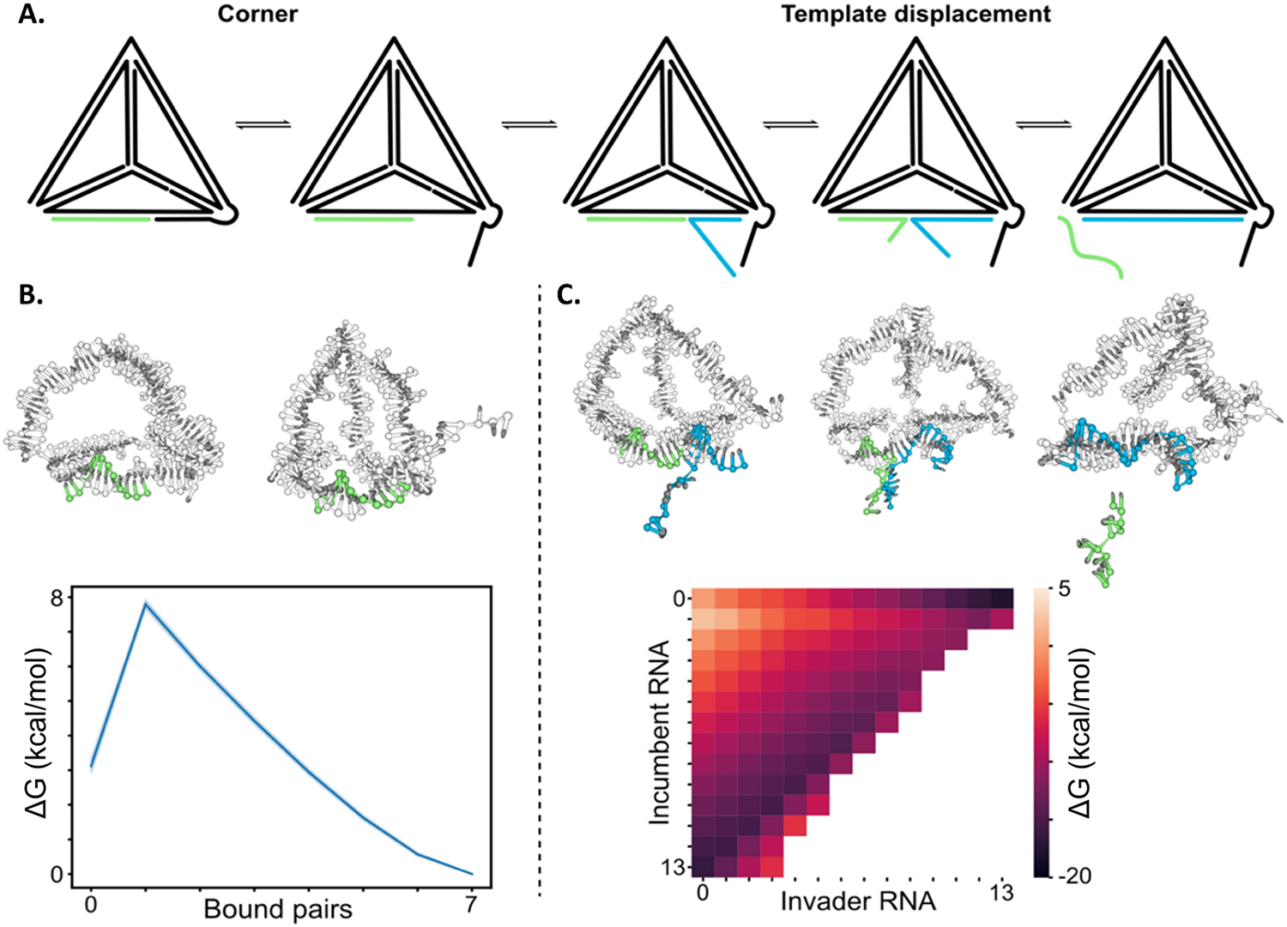
Simulation of the miRNA catalytic cycle. (A) The cleavage of the miRNA-mimic TDN strand creates a 7 nt region that is transiently open and can act as a toehold for a target miRNA (blue strand) to remove the incumbent 13 nt fragment (green strand) by toehold-mediated strand displacement. (B, C) Virtual-move Monte Carlo simulation with the oxNA model allowed for the sampling of the energy landscape for both the availability of the toehold (B) and the strand displacement process (C).

Before the catalytic cleavage step of the target miRNA, the RNA sequence from TDN (miRNA-mimic strand) must first be cleaved by RNase H on the DNA-RNA hybrid. After the cleavage of the TDN miRNA-mimic strand, a 7 nt strand remains hybridized to the TDN, which can potentially reduce the rate of target miRNA binding, by competing with miRNA for TDN binding. Thus, the process of dissociation of the 7 nt strand was evaluated to determine the probability of this dissociation step and consequent availability of the single-strand toehold for the target miRNAs to bind.

To characterize the catalytic mechanism of the TDN, we used a recently developed oxNA model that allows for coarse-grained simulations of DNA-RNA hybrids.^31^ Free energy landscapes were generated using the “virtual-move” Monte Carlo (VMMC) algorithm with umbrella sampling to capture rare events. The free energy landscape resultant from the dissociation of the 7 nt strand (Figure 5B), shows a free energy difference between the bound strand and single-stranded toehold of 3 kcal/mol. This indicates that, at equilibrium, the toehold is expected to be available less than 1% of the time. From this landscape, we also observe that there is an entropic cost for the binding of the first base pair when the 7 nt strand rebinds. Once the miRNA has bound to the toehold, displacement of the 13 nt fragment proceeds via a strand exchange mechanism rather than spontaneous dissociation (Figure 5C). The strand displacement reaction is driven thermodynamically and kinetically by the toehold.

Overall, there is potential to improve the catalytic mechanism of the TDN by increasing the availability of the toehold, by either reducing the binding strength or increasing the entropic barrier.

### 2.5 Evaluation of TDN-amiR-21 Bioactivity

TDN-amiR-21 inhibitory activity was initially assessed using the same luciferase reporter system used for the selection of the anti-miR-21 gapmer sequence. TDN-amiR-21, S1-amiR-21 strand, and the anti-miR-21 gapmer were analyzed 24 h post-transfection (Figure S8). All strands and structures showed miR-21 inhibition, with the free anti-miR-21 gapmer showing the highest activity.

Next, TDN-amiR-21 inhibitory activity was tested in a human glioblastoma cell line (U87), a model with higher relevance in a neural context than the previous reporter model used. For this test, endogenous miR-21 expression was quantified by Quantitative Reverse Transcription-Polymerase Chain Reaction (RT-qPCR) 24 and 48 h after transfection with different concentrations of TDN-amiR-21 and free anti-miR-21 gapmer using Lipofectamine. Additionally, a TDN-amiR-21 with fully PS-modified gapmer sequence (TDN-amiR-21_PS) was also transfected and analyzed. PS modifications were analyzed to verify the influence in the gapmer activity, envisioning the further free delivery of TDN without the transfection reagents, where PS may contribute by increasing resistance against nucleases.^32^ Both TDN types and the anti-miR-21 gapmer sequence induced a concentration-dependent reduction of endogenous miR-21 expression, demonstrating miR-21 inhibitory activity (Figures 6A and S9). The free gapmer showed a tendency for a higher inhibition, with significant differences to TDN-amiR-21 and TDN-amiR-21_PS at the lowest concentration tested (30 nM), although with some variability at this concentration. This tendency was in accordance with the preceding results from luciferase activity. However, at the highest concentrations tested (100 and 300 nM), no significant differences were found between the three conditions, all exhibiting mean downregulation levels above 80% at both tested time points after transfection (24 and 48 h), indicating an equivalent efficacy in inhibiting miR-21 (Figures 6A and S9). In addition, lower variability is observed for the higher concentrations tested, further indicating that robust downregulation can indeed be achieved.

**Figure 6:**
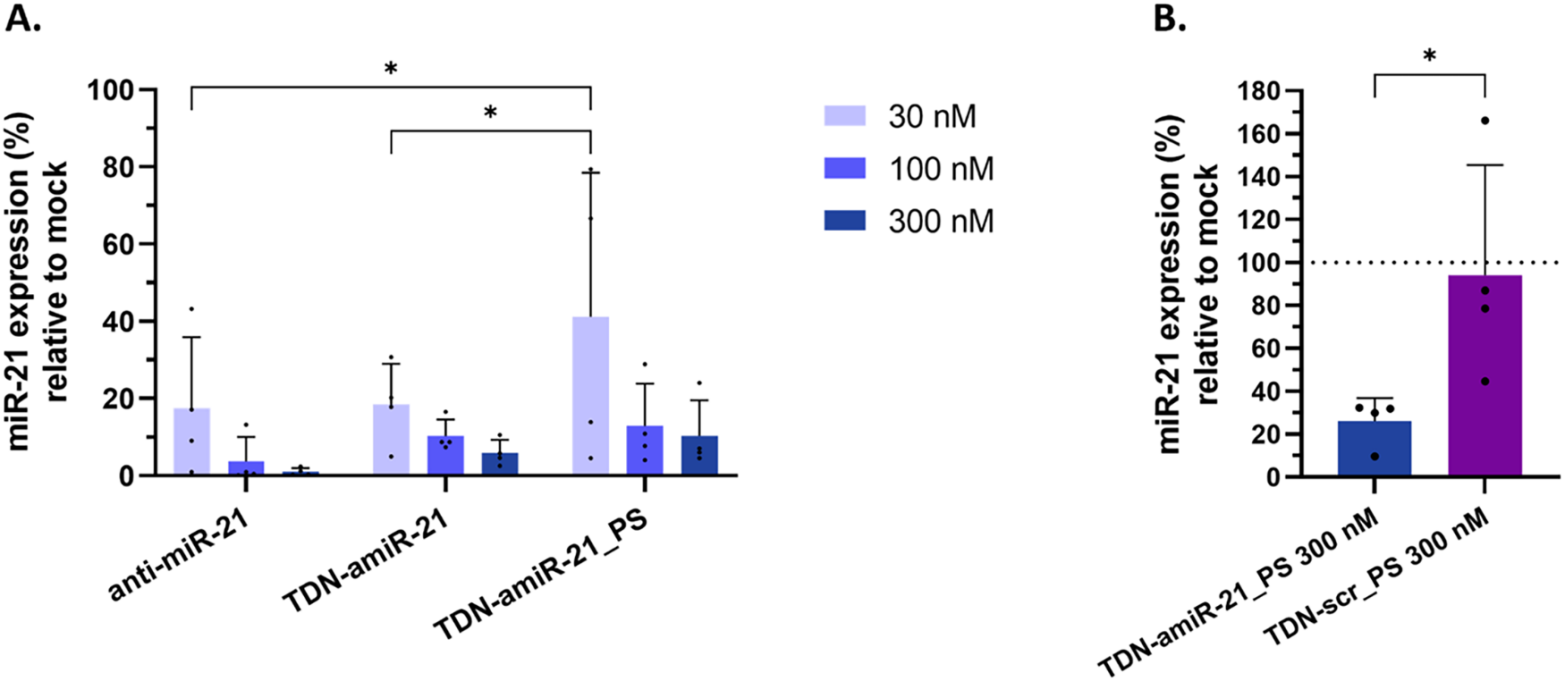
Downregulation of miR-21 quantified by RT-qPCR in U87 cells at 24 h post-transfection of anti-miR-21 gapmer, TDN-amiR-21, and TDN-amiR-21_PS (30, 100, and 300 nM) using Lipofectamine 3000 (A) or TDN-amiR-21_PS and TDN-scr_PS (300 nM each) using Lipofectamine RNAiMAX (B). Mean ± SD from 4 independent experiments (4 biological replicates, with 3 technical replicates each). (A) Repeated measures two-way ANOVA (matching both factors and assuming sphericity), with Tukey’s multiple comparisons test. (B) Two-tailed paired parametric *t* test. *p < 0.05.

To test the specificity of the miR-21 inhibition by TDN-amiR-21_PS, a TDN with a scrambled sequence (TDN-scr_PS) was designed and tested. The same nucleotide modifications of TDN-amiR-21_PS were applied to TDN-scr_PS to capture nonspecific effects. After 24 h, transfection of nanostructures at 300 nM, TDN-amiR-21_PS induced a 74% miR-21 expression reduction, while TDN-scr_PS does not show a significant reduction *versus* mock control, demonstrating the specificity of miR-21 inhibition by TDN-amiR-21_PS. The observed high variability in the TDN-scr_PS does not present a consistent directional change and can be attributed to the multiple high-affinity chemical modifications, whereas the sequence-matched TDN-amiR-21_PS yields a consistent reduction across replicates, significantly different from the control.

### 2.6 TDN-amiR-21 Delivery

To evaluate the potential of TDN as an anti-miR-21 delivery vector, cells were directly incubated with the oligonucleotides without the use of transfection reagents (free uptake), and miR-21 downregulation was evaluated by RT-qPCR.

Initially, different concentrations of TDN-amiR-21_PS and anti-miR-21 were tested 72 h post administration. A reduction in miR-21 expression was obtained at a concentration-dependent level with TDN-amiR-21_PS, while the free anti-miR-21 did not cause a significant reduction of miR-21 (Figure 7A). At 1 μM TDN-amiR-21_PS, the higher downregulation of miR-21 (approximately 73%) was obtained.

**Figure 7:**
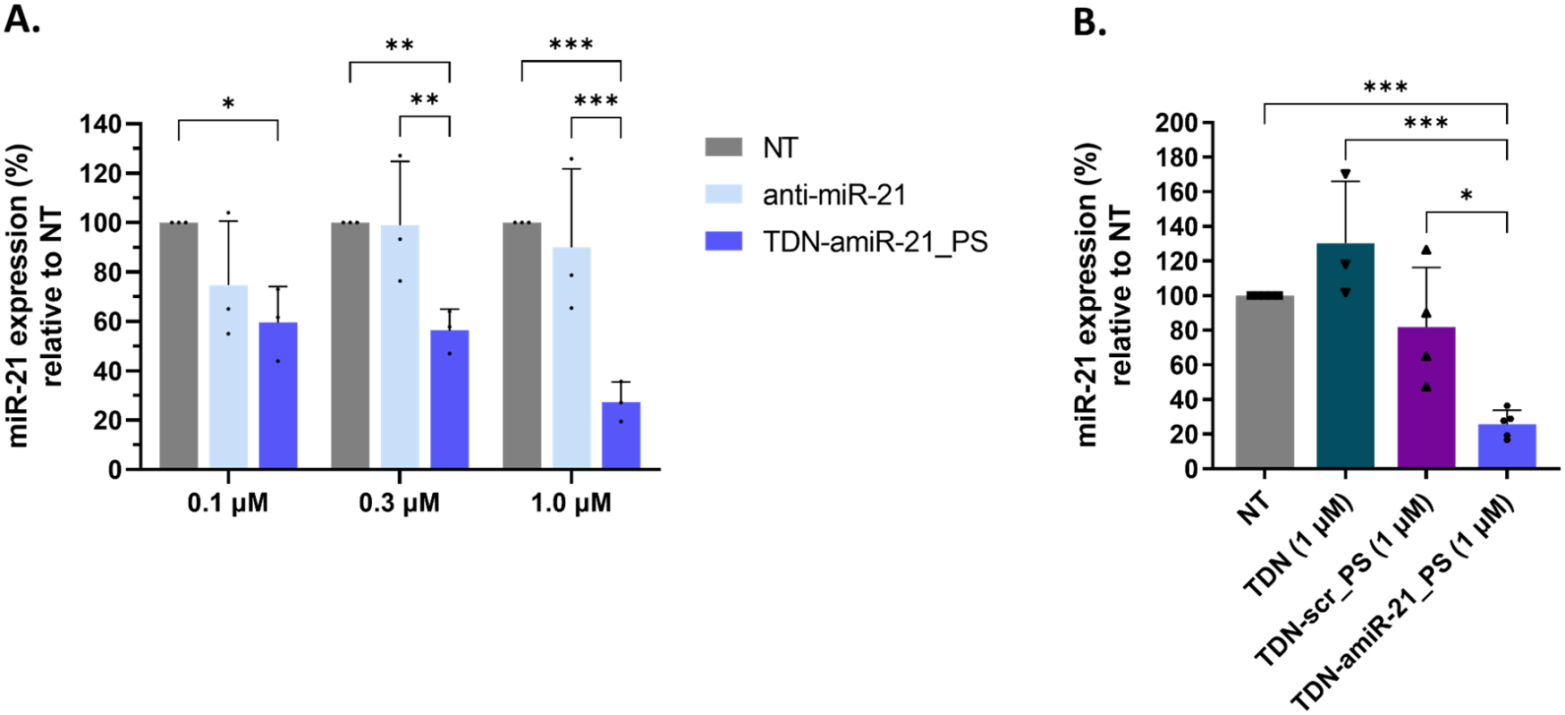
Downregulation of miR-21 quantified by RT-qPCR 72 h after free uptake in U87 cells. (A) Analysis of different concentrations (0.1, 0.3, and 1 μM) of free anti-miR-21 gapmer or TDN-amiR-21_PS. Mean ± SD from 3 independent experiments (3 biological replicates, with 3 technical replicates each). Repeated measures two-way ANOVA (matching both factors and assuming sphericity), with Tukey’s multiple comparisons test. (B) Analysis of different TDN (TDN-amiR-21_PS, TDN-scr_PS, and a regular TDN without nucleotide modifications) at 1 μM. Mean ± SD from at least 3 independent experiments (3 biological replicates, with 3 technical replicates each). Ordinary 1-way ANOVA (assuming sphericity) with Tukey’s multiple comparisons test. *p < 0.05; **p < 0.01; ***p < 0.001.

Next, miR-21 expression was analyzed at different time points (24 to 72 h) after free uptake of 1 μM TDN-amiR-21_PS or anti-miR-21 (Figure S10). Again, a higher miR-21 inhibitory effect of TDN-amiR-21_PS *versus* free anti-miR-21 was verified, with higher differences observed 48–72 h after free uptake. Overall, these results indicate a higher efficiency of anti-miR-21 delivery upon using TDN as a delivery vector. When comparing only the TDN-amiR-21 delivery, there were no significant differences in miR-21 expression between the different time points.

The expression of miR-21 was compared after free uptake of TDN-amiR-21_PS and different control TDNs (scramble TDN-scr_PS and a regular TDN without nucleotide modifications), to verify the miR-21 inhibition specificity after delivery of TDN-amiR-21_PS. Both TDN-scr_PS and the regular TDN did not cause a significant alteration of miR-21 expression, while TDN-amiR-21_PS caused a reduction of approximately 74% in relation to untreated cells, demonstrating the specificity of TDN-amiR-21_PS (Figure 7B). No cytotoxicity was observed for TDN-amiR-21_PS or TDN-scr_PS in relation to untreated cells after 72 h (Figure S11).

Additionally, miR-21 inhibition after free uptake of TDN-amiR-21_PS and TDN-amiR-21 was compared to verify the influence of PS modifications. A similar level of miR-21 inhibition was observed, with no significant differences between the free uptake of TDN-amiR-21_PS and TDN-amiR-21 (Figure S12).

## 3 Discussion

DNA nanostructures have shown potential for drug delivery and especially for therapeutic oligonucleotide delivery. The structural geometry of TDN has been widely explored due to its interactions with cellular membrane, promoting cellular uptake through macropinocytosis, lipid-raft-/caveolae- or clathrin-mediated endocytosis, with macropinocytosis-related protein sorting nexin5 (SNX5), caveolin-1, and scavenger receptors presented as some of the main receptors involved.^10,33–37^

In this study, a new integrated TDN design configuration was explored, focusing on the integration of a gapmer-based antisense oligonucleotide (anti-miR-21) within the TDN framework with the ultimate goal of achieving functional cell delivery. A key motivation for this design is not requiring the use of extensions to load OTs into TDN, preserving possible functionalization sites for future multifunctionalization. Maintaining overall structural integrity and resistance to degradation is also expected.

Few designs of DNA nanostructures have been reported that integrate functionally active OTs sequences within the core scaffold of DNA nanostructures, being part of the structural edges of framework-type DNA nanostructures.^38–40^ Here, we integrate *ab initio* the active sequence in the oligonucleotide strands that form the nanostructure. In addition, we explored a triggering mechanism based on the recruitment of the RNase H enzyme by the active gapmer-based LNA-modified nucleotide sequence region. The active gapmer sequence forms a double strand with a complementary short RNA sequence in the opposite strand, effectively forming one of the structural edges of the TDN containing a DNA:RNA heteroduplex. This design allows in theory the reconfiguration of the structure intracellularly after an initial recognition of the DNA:RNA heteroduplex by the RNase H enzyme, exposing the anti-microRNA sequence and enabling it to bind and inhibit its target microRNA (Figure 5).

The characterization of TDN-amiR-21 demonstrated a successful self-assembly, with approximately 88% of monomeric structures, according to gel densitometry quantification, presenting an approximated mean hydrodynamic diameter of 9 nm, according to DLS. Apparent heights up to 9.68 nm were detected by AFM, including mainly monomeric structures and a few higher-order structures (concatemers). In spite of some possible limitations of DLS for analysis of nonspherical, noncompact, and charged particles, it is still a practical and fast complementary technique for size characterization of DNA nanostructures in solution, having demonstrated a good correlation with AFM results.^41^

Initially, we demonstrated the functionality of our new design by showing that RNase H was efficiently recruited by the gapmer anti-miR-21 within the TDN *in tube*, inducing multiple miR-21 cleavages, indicating its catalytic potential. Formal quantitative turnover analysis remains to be performed to fully characterize the catalytic kinetics experimentally. Additionally, oxNA-based coarse-grained simulations reveal the catalytic potential of TDN-amiR-21 through strand displacement reactions. The simulation of the catalytic mechanism enabled the identification of the availability of the single-strand TDN toehold as the limitation step in the binding of subsequent miRNA, highlighting the potential to thermodynamically optimize the TDN sequence in this region to increase the catalytic mechanism efficiency. These simulations provide important mechanistic insight and theoretical guidance for future optimizations of TDN-amiR-21 design, where each simulated dynamic step can potentially be experimentally demonstrated in the future.

Both TDN-amiR-21_PS and TDN-amiR-21 (active gapmer with or without PS modifications, respectively) showed low degradation in FBS up to 48 h, with the higher degradation occurring after initial contact with FBS. This is derived from partial degradation of strand S4 that included the RNA sequence due to nucleases present in the serum. In the future, a library with different patterns of modifications to the RNA region, as well as shortening of the RNA region, could be tested to increase the resistance of this sequence, maintaining structural stability until intracellular recruitment of RNase H. The gapmer region in S1-amiR-21_PS showed higher resistance to nuclease degradation than that in S1-amiR-21, due to the presence of PS modifications. When strands self-assemble into the TDN-amiR-21 structure, there is higher protection than when strands are free, a characteristic stabilizing effect due to the 3D structural geometry.^3,42^

When miR-21 inhibitory activity was assessed after cell transfection with Lipofectamine, the single-stranded oligonucleotide anti-miR-21 gapmer showed higher activity than TDN-amiR-21 in a luciferase assay (U2OS cells) and a tendency toward higher activity than TDN-amiR-21 by RT-qPCR (U87 cells). The higher efficiency of the single-stranded anti-miR-21 gapmer *versus* TDN-amiR-21 when transfected was not unexpected, as the single-stranded oligonucleotide was free to quickly bind miR-21, contrary to TDN, which initially required RNase H activity to expose the anti-miR-21 sequence. Moreover, the TDN may cause some steric hindrances, delaying the binding to miR-21. In fact, our modeling of miR-21 engagement with the TDN active gapmer region clearly shows that an energy barrier needs to be overcome for the miR-21 target to hybridize with the active gapmer sequence within the TDN. This occurs due to a process of strand displacement that needs to occur starting from a toehold region that is only transiently opening for target RNA binding (Figure 5).

However, when miR-21 inhibitory activity after free uptake of TDN-amiR-21_PS and free anti-miR-21 gapmer is evaluated, higher activity for TDN-amiR-21_PS is observed, in opposition to very low inhibition by free anti-miR-21. This demonstrates a higher efficiency of anti-miR-21 uptake when using TDN as a delivery vector, in comparison to free and gymnotic anti-miR-21 uptake. In addition, we demonstrated the specific anti-miR-21 activity of TDN-amiR-21_PS after free uptake by comparing it with a scrambled TDN (TDN-scr_PS) and a regular TDN with an unrelated structural sequence and without nucleotide modifications. PS have been reported to induce unspecific effects, due to increased affinity toward cellular proteins.^43^ Additionally, the presence of numerous high-affinity nucleotide modifications, such as LNAs in gapmer ASOs, can lead to lower predictability in terms of off-target effects.^44^ Indeed, there is a slight tendency for more variability using the modified TDN-scr_PS in relation to the unmodified regular TDN control. Nonetheless, the anti-miR-21 effect of TDN-amiR-21 was robust and significantly better than all of the controls.

No differences were observed in the miR-21 inhibitory activity of TDN-amiR-21 with or without PS modifications after cell transfection or free uptake. PS modifications in anti-miR-21 oligonucleotides have been reported to increase nuclease resistance and potentiate a higher efficacy in RNase H-mediated degradation, despite a reduction in miR-21 binding affinity.^30,45^ The balance from these effects did not lead to significant differences in miR-21 inhibition under the *in vitro* conditions tested in this study. For future *in vivo* applications, both the number of PS and LNA (or other) modifications must be optimized under the specific conditions to be used, to maintain the increase in nuclease resistance as well as the effectiveness and specificity of miR-21 inhibition.

### 3.1 Limitations and Future Outlooks

This study clearly demonstrates mechanistically RNase H recruitment and miR-21 cleavage by free gapmer anti-miR-21 and TDN-amiR-21 *in tube*. Although the intracellular mechanism of miR-21 inhibition has been functionally validated, the intracellular RNase H dependence remains to be formally demonstrated and investigated in the future. However, the miR-21 inhibition observed from TDN-amiR-21 could potentially arise from (a) RNase H cleavage when/where miR-21 is transiently accessible, free from Ago complex, (b) antisense steric blocking of accessible miR-21 pools, and/or (c) interference with miR-21 biogenesis by binding precursor miRNA-21 (pre-miRNA). Furthermore, trafficking and predominant subcellular localization of TDN-amiR-21 remain to be further investigated in the future.

## 4 Conclusion

Here, a novel TDN design was developed with an antisense oligonucleotide (ASO) anti-miR-21 gapmer sequence fully integrated in the nanostructure framework (TDN-amiR-21). The gapmer mediates an RNase H–dependent cleavage mechanism that results in both structural reconfiguration and target RNA cleavage, thereby triggering a potential catalytic target miRNA degradation. Mechanistic studies, including biochemical RNase H assays and coarse-grained simulations of strand displacement, indicate the potential for catalytic RNase H-mediated cleavage of target miR-21. The *in vitro* efficacy of the TDN-amiR-21 in cells was demonstrated by the specific miR-21 inhibition in a human glioblastoma cell line model, without transfection reagents. As the active oligonucleotide sequence is integrated into the TDN framework, this leaves room to use extension sequences as anchor sites for further functionalization with ligands.

This study encompasses nanostructure design, molecular modeling, *in vitro* characterization with biochemical assays, and *in vitro* cell culture evaluation, giving important insights of the system for future studies using *in vivo* models for therapeutic applications.

The study demonstrates the feasibility of this innovative strategy of framework integration of gapmer ASO in DNA nanostructures, preserving further functionalization sites and enabling efficient cell silencing relative to free ASO. The dynamic design developed here can be applied as a versatile scaffold, opening further possibilities to advance the field of DNA nanotechnology and contributing to novel rational design strategies for enhancing oligonucleotide therapeutics delivery.

## 5 Methods

### 5.1 Oligonucleotides

The sequences of the oligonucleotides used in this study can be found in the Supporting Information (Tables S1–S3). miR-21, control anti-miR-21 (fully 2′-O-methyl (2′-O-Me), three phosphorothioate (PS) linkages at ends), S1-amiR-21_PS and S1-scr_PS sequences, S1t, S2t, S3t, and S4t were synthesized by IDT—Integrated DNA Technologies (Coralville, IA, USA). All other TDN strands and gapmer anti-miR-21 strands were synthesized by Baseclick GmbH (Neuried, Germany) and Ella Biotech GmbH (Fürstenfeldbruck, Germany). All purchased sequences included purification by high-performance liquid chromatography (HPLC).

Detailed descriptions of all experimental procedures and data analysis are provided in the Supporting Information (SI) Appendix, Methods.

*RNase H assay, TDN assembly, Polyacrylamide gel electrophoresis (PAGE), Dynamic light scattering (DLS), Atomic Force Microscopy (AFM), Coarse-grained modeling, Serum stability assay, miR-21 reporter plasmid construction, Cell culture, Luciferase assay, Transfection and free uptake, Quantitative Reverse Transcription-Polymerase Chain Reaction (RT-qPCR), Cell viability, Statistical analysis.*

## Supplementary Material



## Notes

Oligonucleotides sequences used in the study (Tables S1–S3); methods; supporting results: Gapmer anti-miRNA design and characterization (Figures S1–S3); TDN-amiR-21 design (Figures S4 and S5); characterization of TDN-amiR-21 assembly (Figure S6); RNase H cleavage activity on TDN-amiR-21 (Figure S7); and evaluation of TDN-amiR-21 bioactivity in miR-21 inhibition (Figures S8–S12) (PDF)
