# Supplementary material for: Dynamics of an RNase H‑Responsive Tetrahedral DNA Nanostructure for Efficient Intracellular microRNA Inhibition
Source: Bioconjug Chem. 2026 Apr 22;37(5):888–98. doi: 10.1021/acs.bioconjchem.5c00563 (PMC13195572; doi:10.1021/acs.bioconjchem.5c00563)
Supplement: Supplementary file 1 [file bc5c00563_si_001.pdf]

Supporting Information

Dynamics of an RNase H-responsive Tetrahedral DNA Nanostructure for Efficient Intracellular microRNA Inhibition

Ana S. G. Martins[#1,2], Sara D. Reis[#1], Ruxandra Baboi[1], Francisco Furlan[1], João Cortinhas[1], Filomena A. Carvalho[3,4], Nuno C. Santos[3,4], Jonathan Bath[5], Erik Benson[6], Ana P. Pêgo[1,7], Pedro M. D. Moreno[1*]*

1 i3S/INEB - Instituto de Investigação e Inovação em Saúde / Instituto de Engenharia Biomédica, Universidade do Porto, 4200-135 Porto, Portugal

2 FEUP – Faculdade de Engenharia da Universidade do Porto, 4200-465 Porto, Portugal

3 GIMM – Gulbenkian Institute for Molecular Medicine, 1649-028 Lisbon, Portugal

4 Faculdade de Medicina, Universidade de Lisboa, 1649-028 Lisbon, Portugal

5 Kavli Institute for Nanoscience Discovery, University of Oxford, Dorothy Crowfoot Hodgkin Building, Oxford OX1 3QU, United Kingdom

6 Science for Life Laboratory (SciLifeLab), Department of Microbiology, Tumor and Cell Biology, Karolinska Institutet, 171 65 Solna, Sweden.

7 ICBAS - Instituto de Ciências Biomédicas Abel Salazar, Universidade do Porto, 4050-313 Porto, Portugal

#A.S.G.M. and S.D.R. contributed equally to this work as joint first authors.

Table of Contents

1. **Materials** 3
   1.1. Oligonucleotide sequences 3

2. **Methods** 5
   2.1. RNase H assay 5
   2.2. TDN assembly 6
   2.3. Polyacrylamide gel electrophoresis (PAGE) 6
   2.4. Dynamic light scattering (DLS) 8
   2.5. Atomic Force Microscopy (AFM) 8
   2.6. Coarse-Grained Modeling 8
   2.7. Serum stability assay 9
   2.8. miR-21 reporter plasmid construction 9
   2.9. Cell culture 10
   2.10. Luciferase assay 10
   2.11. Transfection and free uptake 11
   2.12. Quantitative Reverse Transcription-Polymerase Chain Reaction (RT-qPCR) 12
   2.13. Cell viability 13
   2.14. Statistical analysis 13

3. **Supplementary Results** 14
   3.1. Gapmer anti-miRNA design and characterization 14
   3.2. TDN-amiR-21 design 17
   3.3. Characterization of TDN-amiR-21 assembly 18
   3.4. RNase H cleavage activity on TDN-amiR-21 19
   3.5. Evaluation of TDN-amiR-21 bioactivity in miR-21 inhibition 20

4. **References** 23

1. Materials

1.1. Oligonucleotide sequences

Table 1. Sequences of micro-RNA-21 (miR-21) and anti-microRNA-21 oligonucleotides (control anti-miR-21 based on Lennox *et al.* [1], and anti-miR-21 gapmers designed and tested in this study: GAPamiR-21_L3+2 (A) and GAPamiR-21_L4+4 (B)).

Oligonucleotide	Sequence (5’ - 3’)
**miR-21**	rUrArG rCrUrU rArUrC rArGrA rCrUrG rArUrG rUrUrG rA
**Control anti-miR-21**	mU*mC*mA* mAmCmA mUmCmA mGmUmC mUmGmA mUmAmA mG*mC*mU*mA
**GAPamiR-21_L3+2 (A)**	+A*mA*mC*+A mU mC +A GT CTG ATA +A mG*mC*+T*mA
**GAPamiR-21_L4+4 (B)**	+A*+A*+C*+A mU mC mA GT CTG ATA mA +G* +C* +T * +A

Notes: DNA = capital letter N; RNA = rN; Phosphorothioate bond = N*; 2’-O-Methyl = mN; locked nucleic acids (LNA) = +N.

Table 2. Oligonucleotides sequences for assembly of TDN-amiR-21, TDN-amiR-21_PS and TDN-scr_PS. TDN-amiR-21 was assembled with S1-amiR-21, S2, S3 and S4. TDN-amiR-21_PS was assembled with S1-amiR-21_PS, S2, S3 and S4. TDN-scr_PS was assembled with S1-scr_PS, S2, S3 and S4-scr.

Oligonucleotide	Sequence (5' - 3')
**S1-amiR-21**	GCC TGC ACA CTT **+A+A+C +AmUmC mAGT CTG ATA mA+G+C +T+A**T TGC CAC CCG CTC GTG CCG CCG TTG GTA CCG CGG
**S2**	CGC GGC GTA ACT TCT GAT GCT TGT GTG CAG GCC CGC GGT ACC TTC GCC GCG TGA CTA GAG CCC G
**S3**	CGG CGG CAC GAG CGG GTG GCT TCG GCG ACC TCA CCC TCC CGC TTC GGG CTC TAG TCA CGC GGC G
**S4**	TAG CTT rArUrC rArGrA rCrUrG rATG TTT TGC ATC AGA AGT TAC GCC GCG TTG CGG GAG GGT GAG GTC GCC G
**S1-amiR-21_PS**	GCC TGC ACA CTT **+A*+A*+C*+A*mU*mC*mA*G*T*C*T*G*A*T*A*mA*+G*+C*+T*+A*** T TGC CAC CCG CTCGTG CCG CCG TTG GTA CCG CGG
**S1-scr_PS**	GCC TGC ACA CTT **+C*+C*+A*+A*mA*mG*mU*C*G*C*C*C*G*A*T*mA*+C*+G*+T*+G** T TGC CAC CCG CTC GTG CCG CCG TTG GTA CCG CGG
**S4-scr**	CAC GTA rUr CrG rGr GrC rGr ArC rUT TGG TTG CAT CAG AAG TTA CGC CGC GTT GCG GGA GGG TGA GGT CGC CG

Notes: DNA= capital letter N; RNA= rN; Phosphorothioate bond = N*; 2'-O-Methyl = mN; locked nucleic acids (LNA) = +N. **Green sequence**: gapmer amiR sequence. **Purple sequence**: scramble sequence.

Table S3. Oligonucleotides for the assembly of a control TDN (based on Goodman *et al.* [2]).

Oligonucleotide	Sequence (5' - 3')
**S1t**	TTT AGG CAG TTG AGA CGA ACA TTC CTA AGT CTG AAA TTT ATC ACC CGC CAT AGT AGA CGT ATC ACC TTT
**S2t**	TTT CTT GCT ACA CGA TTC AGA CTT AGG AAT GTT CGA CAT GCG AGG GTC CAA TAC CGA CGA TTA CAG TTT
**S3t**	TTT GGT GAT AAA ACG TGT AGC AAG CTG TAA TCG ACG GGA AGA GCA TGC CCA TCC ACT ACT ATG GCG TTT
**S4t**	TTT CCT CGC ATG ACT CAA CTG CCT GGT GAT ACG AGG ATG GGC ATG CTC TTC CCG ACG GTA TTG GAC TTT

2. Methods

2.1. RNase H assay

For the RNase H assays with the anti-miR-21 sequences, each anti-miR-21 was mixed with miR-21 at a ratio of 1:1 (3 pmol each) in 140 mM KCl and 3 mM $MgCl_2$, 30 mM Tris-HCl pH 7.4 buffer, and incubated for 30 min at 37°C. Then 2.5 U RNase H enzyme (New England Biolabs, Ipswich, MA, USA) was added to the reaction mixture, followed by an incubation up to 15 min at 37 °C. Enzyme reactions were stopped with 10 mM EDTA. Samples were mixed with loading dye and analyzed by polyacrylamide gel electrophoresis (PAGE).

For the RNase H assays with the TDN integrating anti-miR-21 (TDN-amiR-21), a mix of TDN-amiR-21 : miR-21 was done at a 1:1 ratio (6 pmol each) or a 1:10 ratio (1 pmol TDN-amiR-21 to 10 pmols miR-21) in a 75 mM KCl and 3 mM $MgCl_2$, 30 mM Tris-HCl pH 7.4 buffer. After the addition of RNase H, samples were incubated for different time periods, up to 2 h, at 37 °C. After incubation, samples were mixed with loading dye, and frozen at -20 °C or immediately analyzed by PAGE.

2.2. TDN assembly

The assembly of TDN-amiR-21 and other TDN variations followed the conditions reported by Goodman et al. [2, 3], with novel designed sequences (**Table S2**) and some alterations in buffer and annealing temperatures, as indicated hereafter. TDN assembly was performed by adding equimolar amounts of the four strands at 1 μM, in 15 mM $MgCl_2$, 10 mM Tris-HCl pH 7.4 buffer. Strands were then subjected to the following annealing process: 95 °C, 3 min, followed by 65 °C, 45 min, then 20 °C, 5 min and finally 8 °C, 5 min, with a temperature decreasing rate of 3 °C/s, using a Biometra TPersonal thermocycler (Analytik Jena GmbH, Jena, Germany). For assembly of larger volumes (typically 1 mL), an Eppendorf ThermoMixer C (Eppendorf SE, Hamburg, Germany) was used in the following conditions: 95 °C, 3 min, then 65 °C, 30 min, after which samples were taken to room temperature (RT) for 5 min and finally transferred to ice.

For the assembly of a control TDN (based on Goodman et al. [2]), strands (**Table S3**) were assembled with the following annealing process: 95 °C, 3 min; 75 °C, 45 min; 8 °C, 5 min, with a temperature decreasing rate of 3 °C/s, using a Biometra TPersonal thermocycler (Analytik Jena GmbH), at 20 mM $MgCl_2$, 10 mM Tris-HCl pH 7.4 buffer.

For cellular assays, TDN-amiR-21 and other TDN variations were further concentrated using Amicon Ultra 0.5 mL 10 kDa Centrifugal Filter Units (Merck KGaA, Darmstadt, Germany), with exchange of the TDN assembly buffer to Dulbecco's Modified Eagle Medium (DMEM).

The concentration of nanostructures was verified by measuring the absorbance at 260 nm, using a NanoDrop 1000 spectrophotometer (Thermo Fisher Scientific, Waltham, MA USA), and considering the molar extinction coefficient (ε) as 70% of the total theoretical ε, according to previously reported measurements [4]. TDN assembly was verified by PAGE.

2.3. Polyacrylamide gel electrophoresis (PAGE)

Native PAGE were performed to characterize the TDN-amiR-21 assembly and to evaluate the RNase H assay. Denaturing PAGE was also performed to evaluate the RNase H assay and the serum stability assay.

Polyacrylamide gels were prepared as previously described by us [5]. $MgCl_2.6H_2O$ (EMSURE, Supelco, Merck) was used as supplementation of gels and running buffer when indicated bellow. For the RNase H assay, the anti-miR-21 samples were analyzed in 15% (w/v) native gels (no supplementation), and the TDN-amiR-21 with miR-21, analyzed in either 8% (w/v) native gels (with 3 mM $MgCl_2$ supplementation) or 8-10% (w/v) denaturing gels, with 8 M urea (no additional supplementation). For the TDN-amiR-21 assembly characterization, samples were analyzed in 6% (w/v) resolving gels, supplemented with 5 mM $MgCl_2$. For serum stability assays, 8% (w/v) resolving denaturing gel, with 8 M urea (no additional supplementation), were prepared, and the running buffer (1× TBE) was heated to 45-50 °C before running the gel. All gels were prepared with a top 4% (w/v) stacking gel layer, supplemented as described for the resolving gel concentrations.

For native PAGE, samples were mixed with DNA loading dye (6×) (Thermo Fisher Scientific). For denaturing PAGE, RNA Loading Dye (2×) (New England Biolabs) was added to samples. Then, samples were heated at 95 °C for 3 min and moved to ice for some minutes before loading in the gel (with exception of the gel for RNase H assay, where samples were not heated before gel loading). All gels were run in 1× TBE buffer. A supplement of 3 or 5 mM $MgCl_2$ was added to TBE, when supplementing the gels, as mentioned above.

A GeneRuler Low Range DNA Ladder (Thermo Fisher Scientific) or GeneRuler 100 bp Plus DNA Ladder (Thermo Fisher Scientific) was used as size marker for native gels, and a Low Range ssRNA Ladder (New England BioLabs) was used for denaturing gels.

Gels were stained with SYBR Gold (Invitrogen, Thermo Fisher Scientific), diluted 1:15000 in TBE (1×), for 10 min and revealed in a Gel Doc XR+ Imaging System (Bio-Rad Laboratories, Hercules, CA, USA).

Gel visualization and band quantification were done using Image Lab 6.0.1 software (Bio-Rad Laboratories). Intensity of bands and assembly yield of TDN were quantified by gel band analysis, as previously described by us [5].

2.4. Dynamic light scattering (DLS)

Nanostructures size was determined by DLS in a Zetasizer Nano ZS (Malvern Panalytical, Malvern, United Kingdom), using a quartz cuvette ZEN2112 (Malvern Panalytical). The instrument scattering angle was 173°. Samples were filtered in a 0.22 µm Costar Spin-X column (Corning, Corning, NY, USA) and analyzed at 1 µM, in TDN assembly buffer (15 mM $MgCl_2$, 10 mM Tris-HCl pH 7.4). Three independent measurements were performed at 25 °C, with at least four technical replicates for each sample. Results were analyzed using Malvern Zetasizer software v. 7.13.

2.5. Atomic Force Microscopy (AFM)

Cleaved muscovite mica was used as substrate for imaging TDN-amiR-21. TDN-amiR-21 (5 nM) in Tris-Acetate-EDTA (TAE) 1× buffer were added to the mica surface and allowed to incubate for 5 min. To prevent drying, mica with adsorbed TDN-amiR-21 was washed with filtered TAE 1× buffer, ensuring that the buffer was not completely removed. Quantitative imaging (QI) mode was used in a Nanowizard IV atomic force microscope (Bruker/JPK Instruments, Berlin, Germany) equipped with non-functionalized qp-BioAC CB2 AFM cantilevers (Nanosensors, Neuchâtel, Switzerland). The imaging parameters included a setpoint force of 0.1-0.2 nN, a Z-length of 100 nm, and a pixel time of 5 ms. QI images were acquired with dimensions between 2 µm × 2 µm and 0.3 µm × 0.3 µm, consisting of 256 × 256 pixels. Acquired images were further analyzed using JPK Image Processing software v. 6.0.55 (JPK Instruments).

2.6. Coarse-Grained Modeling

The TDN-amiR-21 structure was assembled as an oxNA model [6] and relaxed using the web service oxview [7]. Since non-standard bases are not supported by the model, all strands were modeled as either DNA or RNA strands. The structures were relaxed, first by a simulation with the 'min' type, and then a second relaxation by molecular dynamics with the max backbone force set to 50.

The availability of the toehold was simulated using VMMC (virtual-move Monte Carlo) using umbrella sampling [8] to bias toward unfavorable states. Order parameters were set up corresponding to the 7 base pairs formed between the corner and the gapmer. The bias weights iteratively

improved until all states were visited with approximately equal probability. The production simulation consisted of 16 replicates that were run for around 200 M steps at 37 °C. After simulation, histograms of the replicate simulations were combined and used to extract a free energy landscape. The relative Gibbs free energy compared to the state where the corner is fully hybridized was calculated by $\Delta G = - RT \ln (K)$, where K is the equilibrium constant, R is the gas constant and T is the absolute temperature.

A similar method was used to simulate the toehold-mediated strand displacement of the incumbent miRNA fragment by a full-length miRNA. The TDN was relaxed in a state where the full-length miRNA was fully bound to the toehold. Order parameters were set up for both the base-pairing of the fragment with the gapmer and the full-length miRNA with the gapmer. Finally, a distance order parameter was set up between the last 4 base pairs of the fragment and the gapmer. After tuning the weights, the simulation was run in 16 replicates for around 180 M timesteps. The unbiased histograms were summed up and the relative Gibbs free energy was calculated comparing to the state where neither strand is hybridized to the gapmer.

2.7. Serum stability assay

Serum stability assay was performed as previously described by us [5], resuspending nanostructures and siRNA samples in DMEM with 10% (v/v) non-inactivated fetal bovine serum (FBS) (Gibco, Thermo Fisher Scientific) (0.3 µM final concentration) and incubating samples at 37 °C. Up to 48 h, sample aliquots of 1.25 pmol were taken at defined time points, immediately mixed with 2× RNA loading buffer (New England Biolabs) and stored at -20 °C, before analyzing by denaturing gel electrophoresis.

2.8. miR-21 reporter plasmid construction

A reporter plasmid of miR-21 expression was built from a dual-reporter NanoLuc/Firefly pmirNanoGlo vector (Promega, Madison, WI, USA) by insertion of a sequence containing four binding sites for miR-21 into the 3' untranslated region (UTR) of the experimental luciferase gene (NanoLuc luciferase). The vector encoded an additional gene (firefly luciferase) with its own promotor, as a control reporter of the vector expression. The DNA sequence cloned into the PmeI-XbaI double digested pmirNanoGlo vector was as follows: 5´-

AAACTAGCGGCCGCTAGTTCAACATCAGTCTGATAAGCTATTCGTCAACATCAGTCTGATAAGCTATTCGTCAACATCAGTCTGATAAGCTATTCGTCAACATCAGTCTGATAAGCTAT -3' (underlined sequence corresponds to miR-21 binding sites; sequence acquired from IDT – Integrated DNA Technologies). Prior to ligation with T4 DNA ligase, the DNA sequence to be cloned was phosphorylated. Ligation products were transformed into DH5α Competent *Escherichia coli* cells (Catalogue #18265017, Thermo Fisher Scientific), and plasmid was purified with QIAGEN Plasmid Plus Maxi Kit (QIAGEN, Venlo, The Netherlands). The engineered plasmid was verified by sequencing.

2.9. Cell culture

Human glioblastoma U87 (ATCC catalogue #HTB-14, kindly provided by Dr. Jorge Lima, i3S-Univ. of Porto) and bone osteosarcoma U2OS (ATCC catalogue #HTB-96) cell lines were cultured in DMEM with high glucose, GlutaMAX and pyruvate (Gibco, Thermo Fisher Scientific), supplemented with 10% (v/v) heat-inactivated (56 °C, 30 min) FBS (Gibco) and 0.1% (v/v) (50 µg/mL) gentamycin (Biowest, Nuaillé, France), and maintained in a humidified incubator at 37 °C with 5% CO2. Inactivated FBS (Gibco) was used in all FBS-supplemented cell culture medium. Cells were passed every 2-3 days and used until 10 passages. Before cell seeding, cell viability was always assessed by trypan blue assay. Cells were routinely tested for mycoplasma contamination by polymerase chain reaction.

2.10. Luciferase assay

U2OS cells were plated at 2.5×10^5 cells/well in 6-well plates. In the next day, cells were transfected with 1 µg plasmid DNA using 3.75 µL Lipofectamine 3000 (Invitrogen) and 1 µL P3000 reagent in a final volume of 2 mL for 24 h. Plasmid-transfected cells were replated into 96-well plates at 1.25×10^4 cells/well. Upon reaching 70-80% confluence, cells were subjected to a second transfection with 3, 10, 30 and 100 nM anti-miR-21 oligos or 100 and 300 nM TDN-amiR-21 and anti-miR-21 oligos, using 0.3 µL Lipofectamine RNAiMAX (Invitrogen), for 24 h, in a final volume of 100 µL. Transfection solutions were prepared in OptiMEM and added to cells in DMEM supplemented with 10% (v/v) FBS and 0.1% (v/v) gentamicin. Cells were lysed and

analyzed for luciferase activity with the Nano-Glo Dual-Luciferase Reporter Assay System (Promega, #N1610). Luminescence of firefly luciferase (FLuc) and NanoLuc luciferase (NLuc) was measured sequentially in a Synergy Mx microplate reader (BioTek Instruments, Winooski, VT, USA), quenching FLuc luminescence before measuring NLuc luminescence. Values of luminescence were first normalized to the control mock transfected cells (treated with empty RNAiMAX), followed by normalization of NLuc luminescence in relation to the luminescence of the FLuc plasmid expression control.

2.11. Transfection and free uptake

For transfection, U87 cells were plated at 2.0 or 3.0 × 10^4 viable cells/well (trypan blue counted) for cell lysis 48 h or 24 h post-transfection, respectively, in 48-well plates. In the next day, cells were transfected with 30, 100 or 300 nM oligonucleotides, using 0.6 µL Lipofectamine 3000 or Lipofectamine RNAiMAX (Invitrogen) in a 200 µL final volume. Transfection solutions were prepared in OptiMEM (Gibco) and added to cells in DMEM supplemented with 10% (v/v) FBS and 0.1% (v/v) gentamicin. At 24 and 48 h post-transfection, cells were washed with PBS and lysed with 250 µL/well RNA lysis buffer from Quick-RNA MicroPrep Kit (Zymo Research, Irvine, CA, USA) for 5 min at RT, followed by a freezing of the plate at -80 ºC. Total RNA was isolated using Quick-RNA MicroPrep Kit (Zymo Research) at RT, including a DNase I (Zymo Research) treatment, according to the kit manufacturer's instructions. Isolated RNA was resuspended in 15 µL nuclease-free water, quantified using NanoDrop 1000 spectrophotometer and stored at -80 ºC.

For carrier-free uptake, U87 cells were plated at 0.8-1.0 × 10^4 viable cells/well in 48-well plates. In the next day, for the experiment evaluating different concentrations and time points, oligonucleotides (100, 300 or 1000 nM) were added to 100 µL final volume and incubated for defined time points (24, 48 and 72 h). At 24 h after free uptake, extra medium was added to prevent evaporation, when continuing incubations. For the experiment evaluating different TDN controls, cell medium was replaced by OptiMEM (Gibco) without serum supplementation and 1000 nM control TDN, TDN-scr_PS (TDN with fully PS modified-scramble gapmer sequence) and TDN-amiR-21_PS (TDN-amiR-21 with fully PS modified-gapmer sequence) were added to cells (100

µL final volume). After 8 h, 20% (v/v) FBS supplemented DMEM (100 µL) was added to cells, to obtain a final concentration of 10% (v/v) FBS, and kept until 72 h after free uptake. At the defined time point, cells were lysed, and RNA was extracted as described above, using Quick-RNA MicroPrep Kit (Zymo Research), quantified using NanoDrop 1000 spectrophotometer and stored at -80 ºC.

2.12. Quantitative Reverse Transcription-Polymerase Chain Reaction (RT-qPCR)

Reverse transcription of 20-40 ng of total RNA isolated from transfection and free uptake experiments was performed using the TaqMan™ MicroRNA Reverse Transcription Kit (Thermo Fisher Scientific, 4366596) and reverse transcription primers (5×) from TaqMan™ MicroRNA Assays (Thermo Fisher Scientific, 4427975, namely, has-miR-21 (ID: 000397) and a reference gene U6 snRNA (a non-coding small nuclear RNA, (ID: 001973)). 1.5 µL of each primer were mixed with the reaction mix and total RNA (2 µL) in a reaction volume of 15 µL and submitted to the standard recommended thermal-cycling (16 °C, 30 min; 42 °C, 30 min; 85 °C, 5 min; 4 °C, 5 min), according to the manufacturer's instructions. The obtained cDNA was stored at -20 °C.

The PCR reaction mixes were prepared using TaqMan Assay (20x) (TaqMan probe and PCR primer set) from TaqMan™ MicroRNA Assays (Thermo Fisher Scientific, 4427975; hsa-miR-21 (ID: 000397) or U6 snRNA (ID: 001973) for miR-21 or U6 amplification, respectively) and TaqMan™ Universal Master Mix II (Thermo Fisher Scientific, 4440043). A mix of cDNA template (0.67 µL) and nuclease-free water (3.84 µL) was prepared and added to the PCR reaction mixes in the PCR plate, obtaining 10.01 µL of total volume of reaction per condition, in accordance with the manufacturer's instructions. For no template controls (NTC) only nuclease-free water was added (4.51 µL). RT-qPCR was carried out on a CFX96 or CFX384 real-time PCR (Bio-Rad Laboratories) system, with the recommended thermal-cycling conditions (95 °C, 10 min, followed by 40 cycles of 95 °C, 15 sec and 60 °C, 60 sec).

Each sample was run in triplicate per RT-qPCR (three technical replicates), and at least three independent experiments were performed (the specific number of independent experiments is indicated in each figure). Results were analyzed using Bio-Rad CFX Maestro 2.3 software (v.

5.3.022.1030). Endogenous expression of miR-21 was quantified and normalized relative to the expression levels of the reference RNA U6, using the ΔΔCT method.

2.13. Cell viability

Cell viability was analyzed using a resazurin-based metabolic activity assay, 72h after free uptake. Cells were incubated for 3 h with 10% (v/v) resazurin solution in regular serum-supplemented medium, and fluorescence (λexc = 530 nm, λem = 590 nm) was measured in a BioTeK Synergy Mx microplate reader.

2.14. Statistical analysis

Statistical analyses were done with GraphPad Prism software v. 9.5.0. Results are presented as mean ± standard deviation (SD), with biological and technical replicates indicated in each corresponding figure. The statistical methods applied subsequently are described in the legends of the corresponding figures. Results were considered statistically significant for a p value < 0.05.

3. **Supplementary Results**

3.1. Gapmer anti-miRNA design and characterization

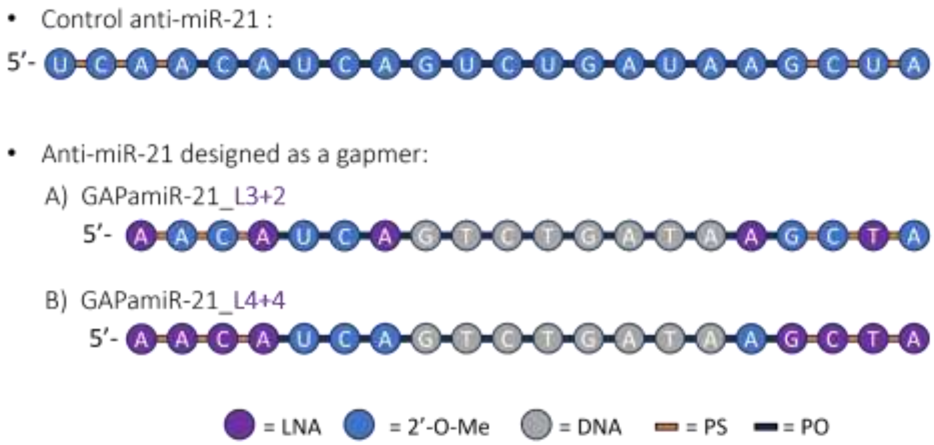

Figure S1. Illustrated sequences and chemical modifications of control and gapmer sequences of anti-miR-21. 2'O-Me: 2′-O-methyl; LNA: locked nucleic acids; PS: phosphorothioate linkage; PO: phosphodiester linkage. The control comprised a fully 2´O-Me modified sequence, with three phosphorothioate (PS) linkages at each end [1]. The gapmer A sequence (GAPamiR-21_L3+2) included 3 alternated LNA at 5'-end flank and 2 LNA at 3'-end flank, while gapmer B (GAPamiR-21_L4+4) included 4 consecutive LNA at each flank. Both gapmers included three PS linkages at each end. Two nucleotides were deleted from the 5'-end of the gapmer sequence in comparison to the control anti-miR-21, to allow the sequence to fit within a fixed TDN edge length of 20 base pairs (bp) since no relevant differences in anti-miR-21 potency were expected in accordance to previously reported results [1].

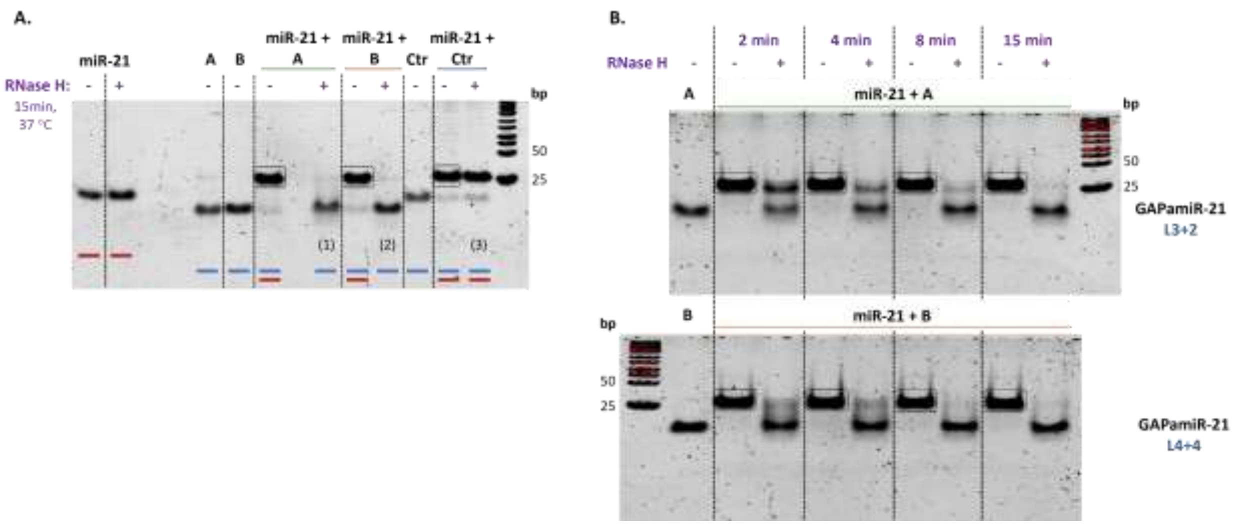

Figure S2. *In vitro* assay to evaluate RNase H recruitment by native PAGE (15%). **A)** Anti-miR-21 gapmers A (GAPamiR-21_L3+2) and B (GAPamiR-21_L4+4) and control anti-miR-21 (Ctr) were hybridized to miRNA-21 ("miR-21 + A", "miR-21 + B" and "miR-21 + Ctr", respectively), leading to the formation of a duplex, and incubated with RNase H for 15 min. Upon 15 min incubation, "miR-21 + A" and "miR-21 + B" show miR-21 cleavage by RNase H (**1, 2**), while "miR-21 + Ctr" does not show RNase H recruitment (**3**). **B)** Duplexes "miR-21 + A" and "miR-21 + B" were incubated for different time points (2, 4, 8 and 15 min) with RNase H.

The addition of RNase H and incubation for 15 min resulted in the dissociation/disruption of both duplexes gapmer A : miR-21 and gapmer B : miR-21 (bands 1 and 2), with observation of a single band with a molecular weight corresponding to gapmer A or B, indicating the recognition by RNase H and consequent miR-21 cleavage/degradation (**Figure S2A**). As expected, no difference was observed for the free miR-21 or the control anti-miR-21 : miR-21 duplex (band 3) after RNase H addition, as RNase H does not act on single or double stranded RNA.

To better determine differences between each gapmer efficiency in RNase H recruitment, an analysis of different time points of RNase H incubation was performed (**Figure S2B**). Gapmer A : miR-21 and gapmer B : miR-21 duplexes were then incubated with RNase H for 2, 4, 8 or 15 min. After a 2-min incubation with RNase H, duplexes with gapmer B were mostly dissociated, thus miR-21 cleaved, while an 8 min-incubation was needed for gapmer A duplexes to be dissociated, demonstrating a higher efficiency of gapmer B in this assay.

The anti-miR-21 gapmers inhibitory activity was then evaluated *in vitro* using a luciferase reporter system in U2OS cell line, which endogenously expresses high levels of miR-21. For that, a pmirGLO luciferase

expression plasmid was constructed by addition of four binding sites for miR-21 in the 3'-untranslated region (UTR) of the luciferase gene. The luciferase-miR-21 reporter plasmid was transfected into U2OS cells, where endogenous miR-21 hybridized to the luciferase mRNA UTR, repressing luciferase expression. A second transfection of U2OS cells (already expressing the reporter plasmid) was done with the anti-miR-21 sequences, expecting to inhibit endogenous miR-21 and, consequently, lead to an upregulation of luciferase expression and activity. In this way, the efficiency of the inhibitory activity of anti-miR-21 sequences can be measured through the increase of luciferase activity (luminescence release) (**Figure S3A**).

The inhibitory activity was analyzed 24 h post-transfection with anti-miR-21 oligonucleotides (**Figure S3**). All sequences led to a concentration-dependent increase of luminescence, which translates an increase on luciferase activity and on miR-21 inhibitory activity, compared to mock transfected cells (**Figure S3B**). Both anti-miR-21 gapmers led to higher luminescence values than the control anti-miR-21 for the highest concentration tested, demonstrating a higher potency/efficiency to inhibit miR-21 than the anti-miR-21 control, in these conditions. No difference was observed between the two anti-miR-21 gapmers. Given the fast RNase H recruitment in the RNase H assay by PAGE (**Figure S2B**), anti-miR-21 gapmer B was the sequence selected to be incorporated into the tetrahedron DNA nanostructure.

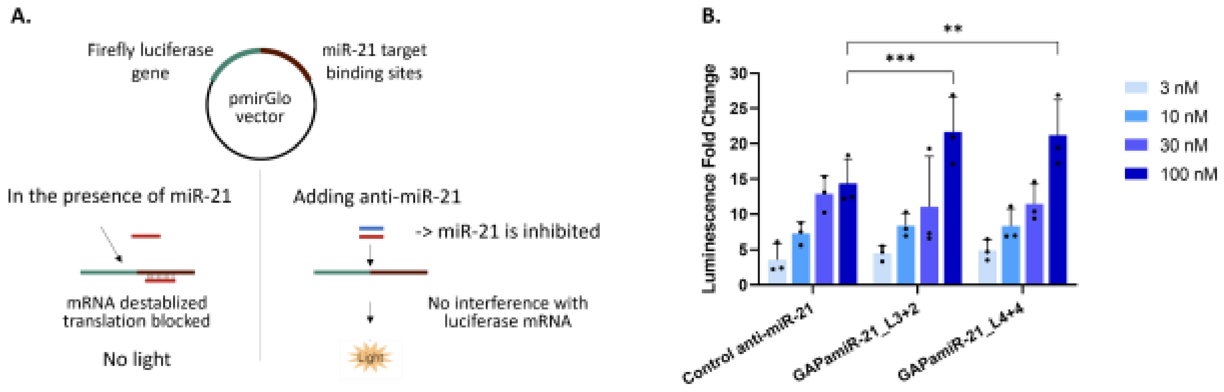

Figure S3. Luciferase assay. **A)** Illustration of the assay mechanism using the pmirGLO vector (image adapted from a Promega illustration). In the presence of endogenous miRNA-21, it will bind to the mRNA of luciferase, blocking luciferase translation, so no luminescence is detected. When transfecting anti-miR-21, the endogenous miR-21 will be suppressed, not interfering with the mRNA of luciferase, so the luciferase protein is translated, which can be detected by the release of luminescence. **B)** Efficiency of anti-miR-21 oligonucleotides (3-100 nM) activity 24 h post-transfection in relation to a mock (empty lipofectamine vector) control, in U2OS cells (human bone osteosarcoma) expressing a luciferase-miR-21 reporter plasmid, detected by luminescence measurement. Mean ± SD from 3 independent experiments, 2

biological replicates each. Repeated measures two-way ANOVA (matching both factors and assuming sphericity), with Tukey's multiple comparisons test. ** $p < 0.01$; *** $p < 0.001$.

3.2. TDN-amiR-21 design

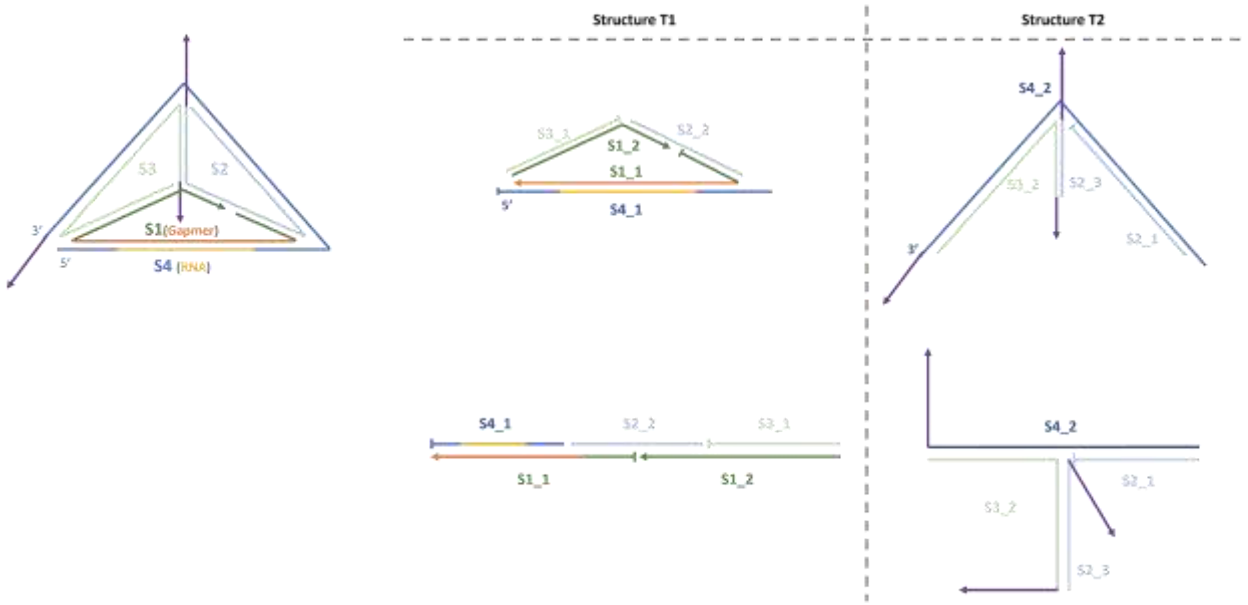

Figure S4. TDN deconstruction in 2 sections (structure T1 and structure T2) to obtain unpseudoknotted structures that could be used for sequence design using NUPACK software. The two sections were analyzed in the same "target test tube" by NUPACK to obtain the sequences with the lowest ensemble defect [9, 10]. Settings for sequence design were defined to 37 °C, with 2 mM $MgCl_2$ and 150 mM NaCl, with maximum ensemble defect of 5.0 %, and preventing the following sequence patterns from the output: CCCC, GGGG, CCCCC, GGGGG, AAAAA, TTTTT. Although only TDN without extensions were used in this study, three overhang extensions were included in the TDN-amiR-21 design stage for possible future TDN-amiR-21 multi-functionalization with ligands.

3.3. Characterization of TDN-amiR-21 assembly

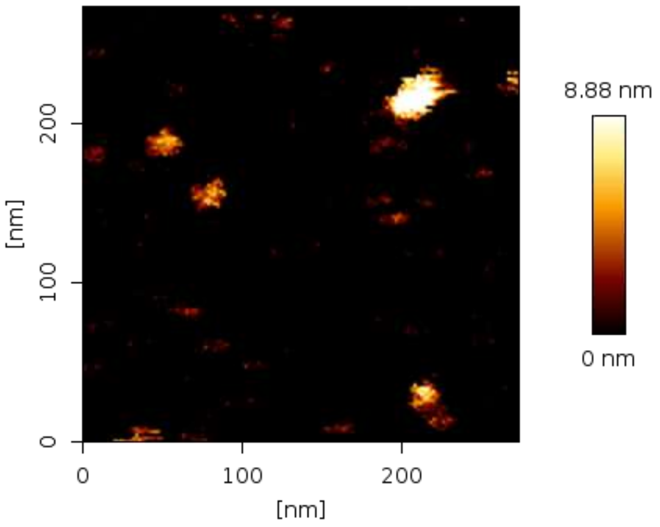

Figure S5. Additional illustrative AFM image, color-coded according to the height of the detected nanostructures (height scale bar in the right). The larger bright structure at the top right is identified as a concatemer, and the three smaller structures identified as TDNs.

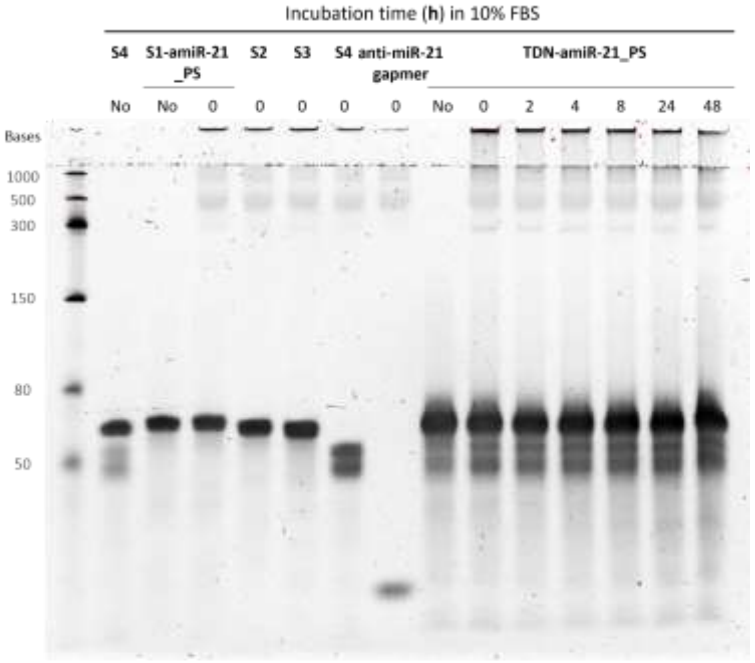

Figure S6. Denaturing PAGE analysis of TDN-amiR-21_PS incubated up to 48 h in DMEM with 10% FBS (non-inactivated). 8% (w/v) resolving denaturing gel, with 8 M urea.

Along the characterization of TDN-amiR-21 with PAGE, different polyacrylamide gel percentages were used to highlight differences in TDN size (e.g. 6% native gel) or focus in both TDN-amiR-21 and miR-21 (e.g. 8% native gel), including 3-5 mM magnesium supplementation in native gels to help in TDN stabilization. Migration of TDN is dependent on salts composition and polyacrylamide percentage, which leads to differences in the apparent molecular weight relative to the ladder (dsDNA) in the diverse gels.

3.4. RNase H cleavage activity on TDN-amiR-21

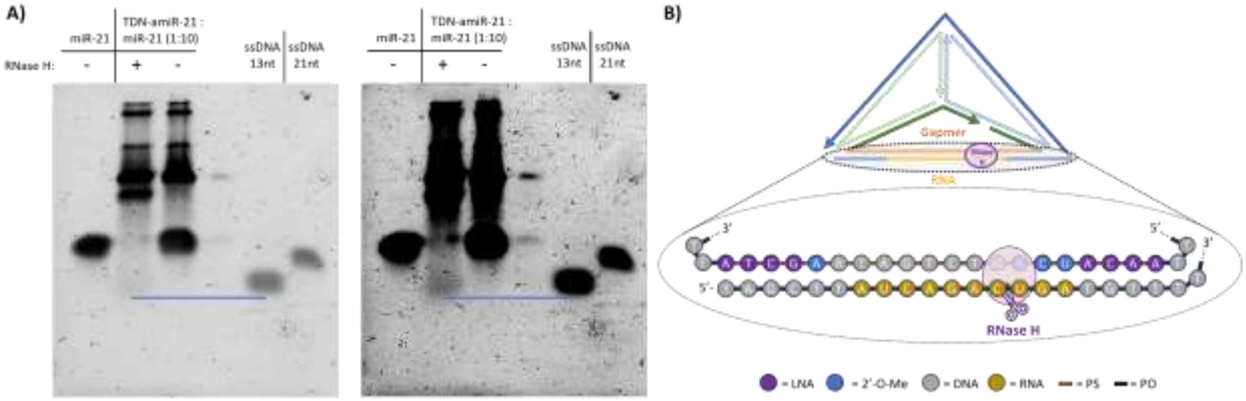

Figure S7. A) Characterization of digestion products after TDN-amiR-21:miR-21 incubation with RNase H by denaturing PAGE (10%) analysis, with 8 M urea supplementation. TDN-amiR-21 was mixed with miR-21 at a ratio of 1:10 and incubated in the absence or presence of RNase H (5 U) for 2 h. Single stranded DNA markers of precise length (13 and 21 nt) were used to compare migration patterns with miR-21 digestion products. Right gel is a high contrast image to enhance the visualization of the digestion products. For TDN-amiR-21:miR-21 treated with RNase H, a noticeable small MW band appears with the same migration as the ssDNA 13 nt marker, indicating that a major product of the miR-21 digestion by RNase H is of similar size (13 nt). **B)** Simplified schematic representation of the assembled TDN sequences, highlighting the RNase H main cleavage site in the active gapmer:RNA duplex region and the nucleotide modifications used in the design.

3.5. Evaluation of TDN-amiR-21 bioactivity in miR-21 inhibition

Cells transfected with 100 or 300 nM TDN-amiR-21 exhibited a 2- to 8-fold increase in luciferase expression, in a concentration-dependent manner (**Figure S7**). Anti-miR-21 gapmer (300 nM) showed the higher miR-21 inhibition, with a 15-fold increase in luciferase expression in relation to non-transfected cells, while the S1-amiR-21 strand (300 nM) resulted in a 10-fold increase. This was an expected result to some extent, as the anti-miR-21 gapmer was freely available to bind to miR-21, while S1-amiR-21, being a longer strand, could be forming some secondary structures that might delay the recognition of miR-21. For TDN-amiR-21, there is one additional step, with structural rearrangement of the TDN, before the anti-miR-21 sequence becomes accessible to miR-21 binding, which can play some influence in the binding affinity and kinetics. Additionally, the free anti-miR-21 gapmer presented PS modifications, while TDN-amiR-21 and S1-amiR-21 did not, only having phosphodiester (PO) bonds. The oligonucleotide backbone substitutions of PO with PS in the anti-miR-21 oligonucleotides could have potentiated a higher efficacy in RNase H-mediated degradation, as suggested by previous studies [11,12].

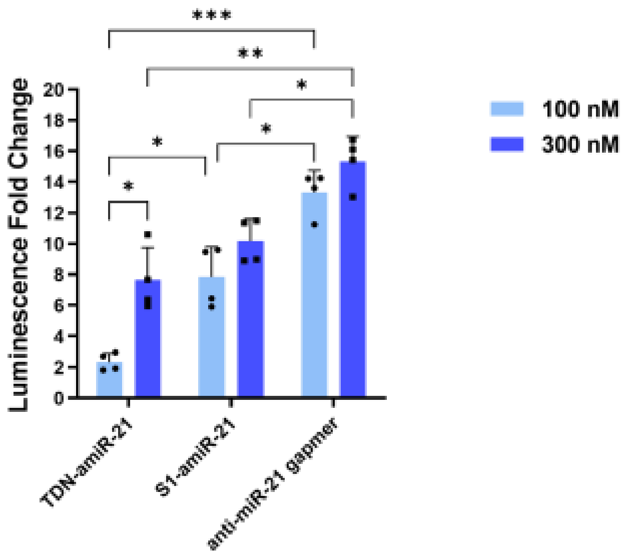

Figure S8. Luciferase activity analyzed 24 h post-transfection with TDN-amiR-21, S1-amiR-21 strand, anti-miR-21 gapmer (100 and 300 nM). Fold change *vs*. non-transfected mock control cells (empty Lipofectamine). Mean ± SD from 4 independent experiments (8 biological replicates each). Repeated measures two-way ANOVA (matching both factors and assuming sphericity), with Tukey's multiple comparisons test. * $p < 0.05$; ** $p < 0.01$; *** $p < 0.001$.

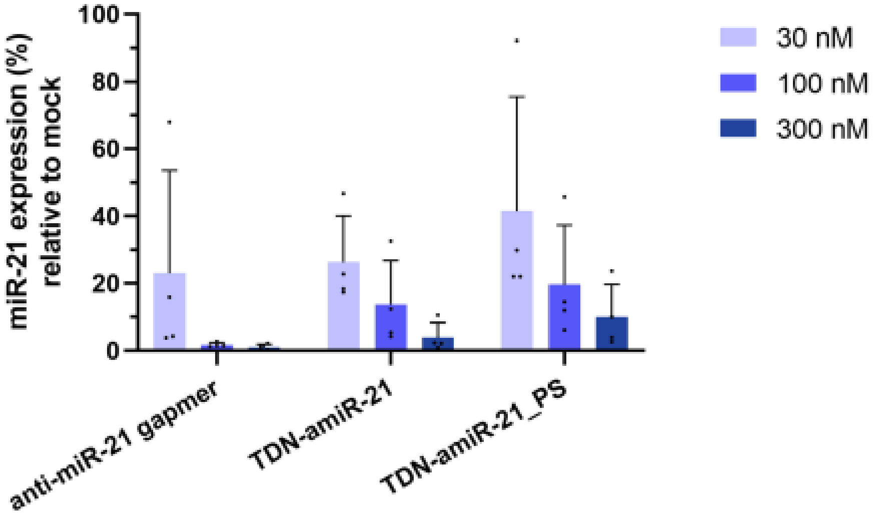

Figure S9. Downregulation of miR-21 quantified by RT-qPCR in U87 cells 48 h post-transfection of anti-miR-21 gapmer, TDN-amiR-21 and TDN-amiR-21_PS using Lipofectamine 3000. Mean ± SD from 4 independent experiments (4 biological replicates, with 3 technical replicates each). Repeated measures two-way ANOVA (matching both factors and assuming sphericity), with Tukey's multiple comparisons test.

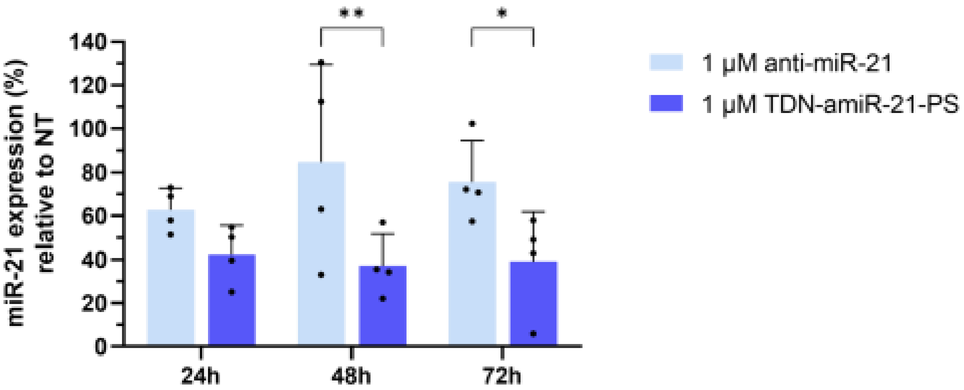

Figure S10. Analysis of miR-21 downregulation 24 h, 48 h and 72 h after free uptake of 1 µM anti-miR-21 and TDN-amiR-21_PS. Mean ± SD from 4 independent experiments (4 biological replicates, with 3 technical replicates each). Repeated measures two-way ANOVA (matching both factors and assuming sphericity), with Tukey's multiple comparisons test. * $p < 0.05$; ** $p < 0.01$.

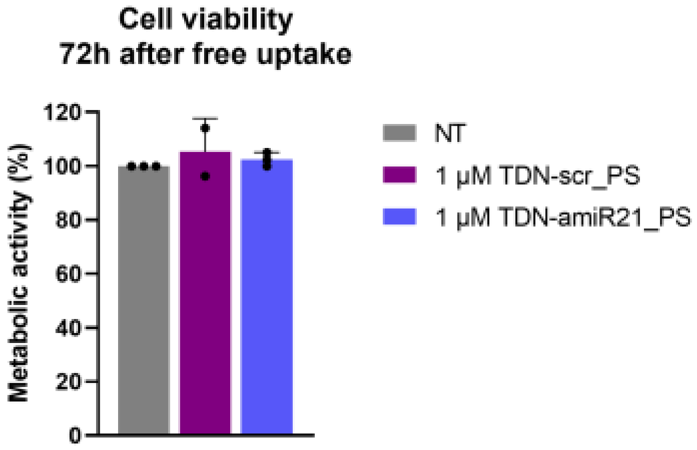

Figure S11. Analysis of cell viability at 72 h after free uptake of 1 µM TDN-amiR-21_PS and TDN-scr_PS. Mean ± SD from 3 independent experiments (3 biological replicates), except for TDN-scr-PS with 2 independent experiments (2 biological replicates). Mixed effects analysis with Tukey's multiple comparisons test.

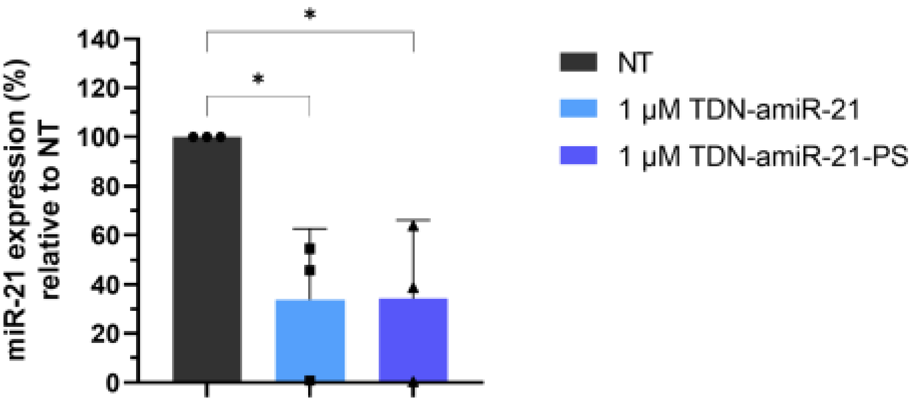

Figure S12. Analysis of the influence of PS modification on TDN activity (cell delivery of anti-miR-21 and miR-21 inhibition) 72 h after free uptake of 1 µM TDN-amiR-21 and TDN-amiR-21_PS. Mean ± SD from 3 independent experiments (3 biological replicates, with 3 technical replicates each). Repeated measures one-way ANOVA (assuming sphericity), with Tukey's multiple comparisons test. * $p < 0.05$.

4. **References**

(1) Lennox, K. A.; Owczarzy, R.; Thomas, D. M.; Walder, J. A.; Behlke, M. A. Improved Performance of Anti-miRNA Oligonucleotides Using a Novel Non-Nucleotide Modifier. *Mol. Ther. Nucleic Acids* **2013**, *2* (8), e117. DOI: 10.1038/mtna.2013.46.

(2) Goodman, R. P.; Schaap, I. A.; Tardin, C. F.; Erben, C. M.; Berry, R. M.; Schmidt, C. F.; Turberfield, A. J. Rapid chiral assembly of rigid DNA building blocks for molecular nanofabrication. *Science* **2005**, *310* (5754), 1661-1665. DOI: 10.1126/science.1120367.

(3) Goodman, R. P.; Berry, R. M.; Turberfield, A. J. The single-step synthesis of a DNA tetrahedron. *Chem. Commun.* **2004**, (12), 1372-1373. DOI: 10.1039/B402293A.

(4) Xing, S.; Jiang, D.; Li, F.; Li, J.; Li, Q.; Huang, Q.; Guo, L.; Xia, J.; Shi, J.; Fan, C.; et al. Constructing Higher-Order DNA Nanoarchitectures with Highly Purified DNA Nanocages. *ACS Appl. Mater. Interfaces* **2015**, *7* (24), 13174-13179. DOI: 10.1021/am505592e.

(5) Martins, A. S. G.; Reis, S. D.; Benson, E.; Domingues, M. M.; Cortinhas, J.; Vidal Silva, J. A.; Santos, S. D.; Santos, N. C.; Pêgo, A. P.; Moreno, P. M. D. Enhancing Neuronal Cell Uptake of Therapeutic Nucleic Acids with Tetrahedral DNA Nanostructures. *Small* **2024**, *20* (29), 2309140. DOI: 10.1002/smll.202309140.

(6) Ratajczyk, E. J.; Šulc, P.; Turberfield, A. J.; Doye, J. P. K.; Louis, A. A. Coarse-grained modeling of DNA–RNA hybrids. *J. Chem. Phys.* **2024**, *160* (11), 115101. DOI: 10.1063/5.0199558.

(7) Bohlin, J.; Matthies, M.; Poppleton, E.; Procyk, J.; Mallya, A.; Yan, H.; Šulc, P. Design and simulation of DNA, RNA and hybrid protein–nucleic acid nanostructures with oxView. *Nat. Protoc.* **2022**, *17* (8), 1762-1788. DOI: 10.1038/s41596-022-00688-5.

(8) Sengar, A.; Ouldridge, T. E.; Henrich, O.; Rovigatti, L.; Šulc, P. A Primer on the oxDNA Model of DNA: When to Use it, How to Simulate it and How to Interpret the Results. *Front. Mol. Biosci.* **2021**, *8*, 693710. DOI: 10.3389/fmolb.2021.693710.

(9) Dirks, R. M.; Lin, M.; Winfree, E.; Pierce, N. A. Paradigms for computational nucleic acid design. *Nucleic Acids Res.* **2004**, *32* (4), 1392-1403. DOI: 10.1093/nar/gkh291.

(10) Wolfe, B. R.; Pierce, N. A. Sequence Design for a Test Tube of Interacting Nucleic Acid Strands. *ACS Synth. Biol.* **2015**, *4* (10), 1086-1100. DOI: 10.1021/sb5002196.

(11) Miroshnichenko, S. K.; Patutina, O. A.; Burakova, E. A.; Chelobanov, B. P.; Fokina, A. A.; Vlassov, V. V.; Altman, S.; Zenkova, M. A.; Stetsenko, D. A. Mesyl phosphoramidate antisense oligonucleotides as an alternative to phosphorothioates with improved biochemical and biological properties. *Proc. Natl. Acad. Sci. U.S.A.* **2019**, *116* (4), 1229-1234. DOI: 10.1073/pnas.1813376116.

(12) Crooke, S. T.; Lemonidis, K. M.; Neilson, L.; Griffey, R.; Lesnik, E. A.; Monia, B. P. Kinetic characteristics of Escherichia coli RNase H1: cleavage of various antisense oligonucleotide-RNA duplexes. *Biochem. J.* **1995**, *312* (2), 599-608. DOI: 10.1042/bj3120599.
